# Novel attempt at discrimination of a bullet-shaped siphonophore (Family Diphyidae) using matrix-assisted laser desorption/ionization time of flight mass spectrometry (MALDI-ToF MS)

**DOI:** 10.1038/s41598-021-98724-z

**Published:** 2021-09-24

**Authors:** Nayeon Park, Jisu Yeom, Raehyuk Jeong, Wonchoel Lee

**Affiliations:** 1 Laboratory of Biodiversity, Department of Life Science, Hanyang University, Seoul, 04763 South Korea; 2 Big Data Biology Lab, Department of Life Science, Chung-Ang University, Seoul, 06974 South Korea

**Keywords:** Ecology, Zoology, Ecology, Ocean sciences

## Abstract

One major difficulty in identifying the gelatinous bodied bullet-shaped Siphonophore, Diphyids, is that their shape is deformed following ethanol fixation. Ethanol often is preferred over other fixatives, since samples fixed in ethanol can be used for molecular studies that can supplement morphological findings. To overcome this problem, we obtained protein mass spectra of ten species of Diphyidae found in the waters of the Kuroshio Current (Northwest Pacific and South Coast of South Korea) to test whether MALDI-ToF MS could be used as a methodology for species identification. In addition, a number of morphological characteristics that can be used with ethanol-treated samples was summarized. Concatenated phylogenetic analysis was also performed to determine the phylogenetic relationship by obtaining partial sequences of four genes (mtCOI, 16S rRNA, 18S rRNA, and ITS regions). Based on our integrative analysis, MALDI-ToF MS was evaluated as a potentially fast, inexpensive, and accurate tool for species identification along with conventional morphological and DNA barcoding for Diphyidae.

## Introduction

Over 60% of Siphonophorae Eschscholtz, 1829 which form unique colonies^1^ through their life are Diphyidae Quoy & Gaimard, 1827^2^. The family Diphyidae consists of 45 species belonging to eight genera, and it is the most species-diverse family of seven belonging to the suborder Calycophorae Leuckart, 1854. Aside from the currently valid 45 species, there is a handful of species that is considered species inquirenda, which require taxonomic review. Through alternation of generations, Diphyidae goes through polygastric (creating eudoxid phase through asexual reproduction) and eudoxid (creating polygastric phase through sexual reproduction) phases^3^. Polygastric Diphyidae consists of one or two nectophores, of which the small bullet-shaped anterior nectophore contains key information for classification, such as somatocyst, mouthplate, ostial teeth, hydroecium, and longitudinal ridges. In fact, there are many instances where the morphological record of Diphyidae often is dependent on the anterior nectophore. Despite their morphological importance for species identification, anterior nectophores often are easily damaged during fixation, since they consist of gelatin. In particular, using ethanol as a fixative will induce dehydration, shriveling the shape of the species to a point that complicates species identification. For this reason, much effort has been asserted in the last decade to utilize DNA barcoding for species identification. However, DNA barcoding is time consuming and costly with many limitations, such as requiring prior knowledge of a target species to design species-specific primers to maximize sequencing success.

In species identification for pathogens or microbes, much easier and faster results can be achieved through Matrix-Assisted Laser Desorption/Ionization Time of Flight Mass Spectrometry (MALDI-ToF MS). MALDI-ToF MS is a method developed by Karas & Hillenkamp^4^, which uses an aromatic carboxylic acid matrix to crystalize the protein, which is then hit by a laser in a vacuum to cause ionization. The ionized particles are passed through a ToF analyzer, where the mass of the protein is calculated by measuring the time of flight^5^. These protein mass spectra serve as unique fingerprints, enabling species identification. Recent studies have been expanded to apply to eukaryotes, such as mosquitoes, fruit flies, sand flies, fishes, and copepods^6–11^. This method has been attempted on jellyfishes as well^12–14^, but it was used to target specific proteins of nematocysts, not to identify species.

**Figure 1:**
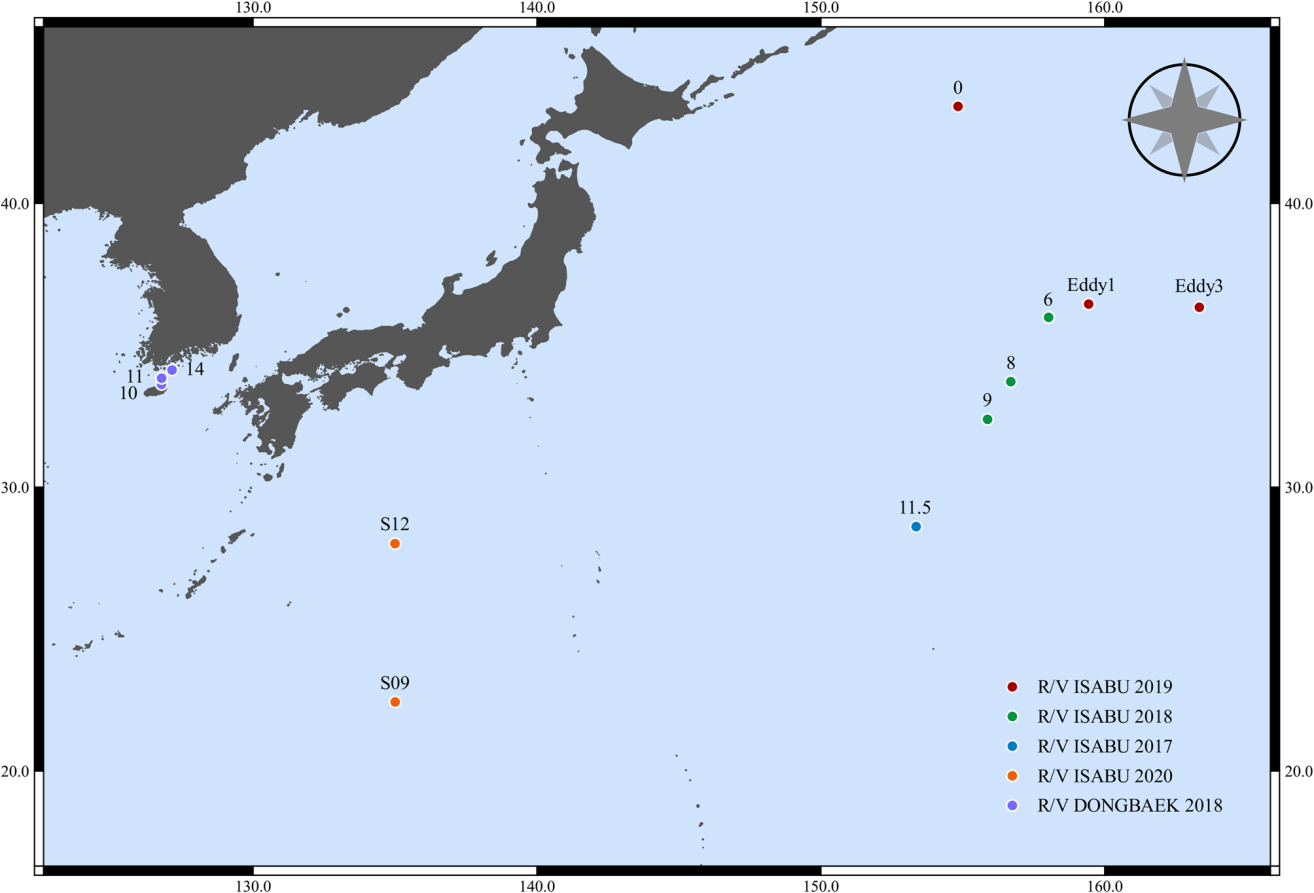
Map of collecting stations marked with the spots.

In this study, we evaluated the potential of MALDI-ToF MS as a tool for species identification of jellyfish by testing the method on ten species of Diphyidae found in the Kuroshio Current and off the coast of Korea (Fig. 1). This was the first case in which MALDI-ToF MS was utilized as a tool for jellyfish species identification. In addition, we conducted an integrated taxonomic review of the ten species based on morphological analysis and molecular analysis of four DNA marker sequences (mtCOI, 16S rRNA, 18S rRNA, and ITS regions). The three integrative approaches utilized in this study will heighten our understanding of bullet-shaped Siphonophore, which has been subject to identification difficulties due to morphological ambiguity.

## Results

### Morphological comparison

**Figure 2:**
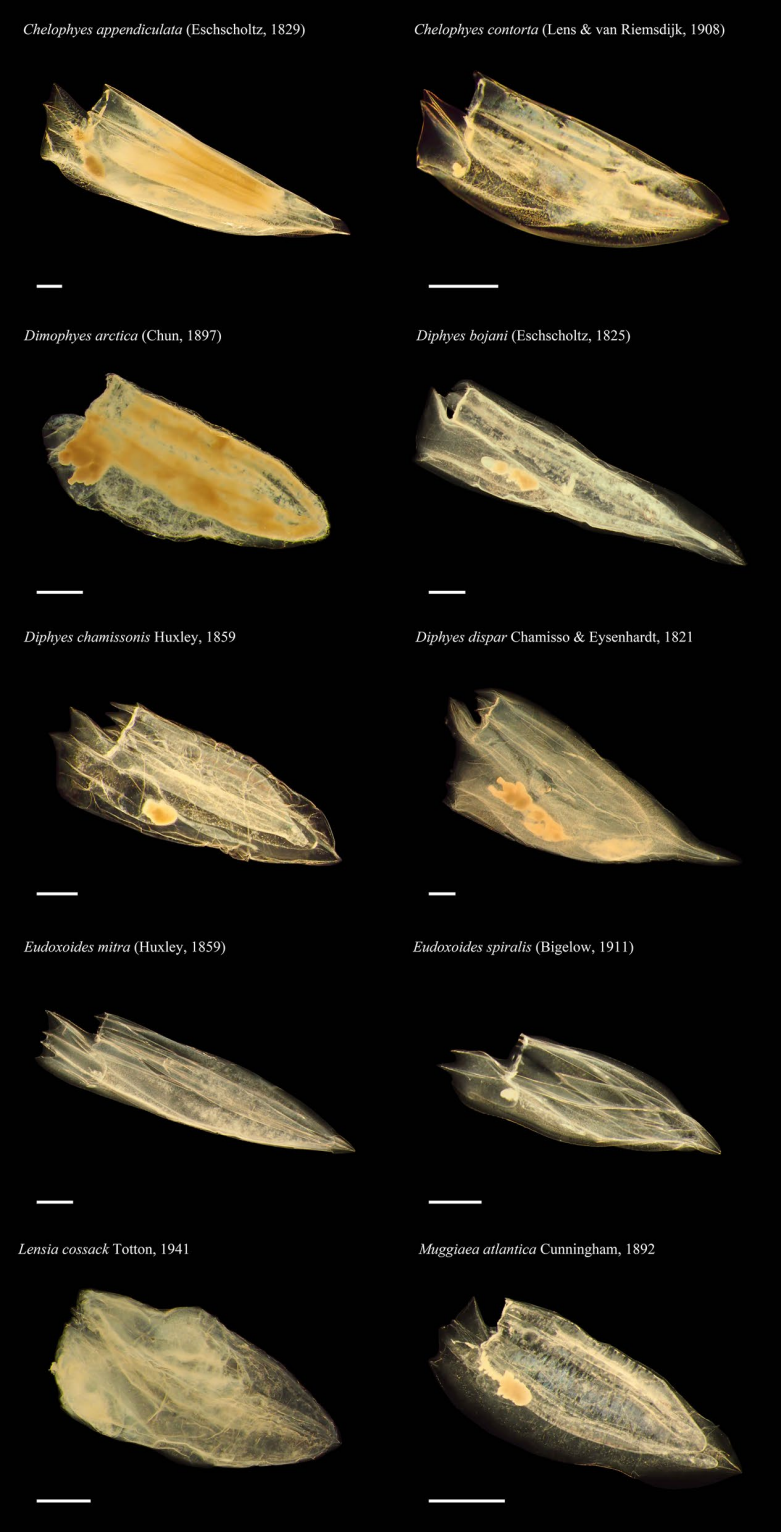
Digital images of anterior nectophores from target Diphyids. All specimens are ethanol fixed. Scale bar = 1 mm.

A total of ten species of Diphyidae was obtained, and all species were recorded using anterior nectophore (Fig. 2). All specimens were bullet-shaped, except for *Diphyes dispar* Chamisso & Eysenhardt, 1821, and *Eudoxoides spiralis* (Bigelow, 1911), which had modified forms of triangular bullet-shaped and twisted bullet-shaped, respectively. Morphological key features such as shape of the apex, number of longitudinal ridges, hydroecium, ostial teeth, mouthplate, and somatocyst were compared (Table 1).

**Table 1:** Morphological comparisons between the ten Diphyidae species from ethanol-fixed samples. O: presence; X: absence.

Family	Diphyidae									
Genus	*Chelophyes*		*Dimophyes*	*Diphyes*			*Eudoxoides*		*Lensia*	*Muggiaea*
Species	*appendiculata*	*contorta*	*arctica*	*bojani*	*chamissonis*	*dispar*	*mitra*	*spiralis*	*cossack*	*atlantica*
Author	Eschscholtz, 1829	Lens & van Riemsdijk, 1908	Chun, 1897	Eschscholtz, 1825	Huxley, 859	Chamisso & Eysenhardt, 1821	Huxley, 1859	Bigelow, 1911	Totton, 1941	Cunningham, 1892
Number of Nectophores	2	2	2	2	1	2	2	1	1	1
Number of Nectophore Types	2	2	2	2	1	2	2	1	1	1
Comparison of Nectophore Size	Anterior > Posterior	Anterior > Posterior	Anterior > Posterior	Anterior ≥ Posterior	X	Anterior > Posterior	Anterior > Posterior	X	X	X
Shape of Anterior Nectophore	Bullet-shape	Bullet-shape	Bullet-shape	Bullet-shape	Bullet-shape	Triangular Bullet-shape	Bullet-shape	Twisted Bullet-shape	Bullet-shape	Bullet-shape
Shape of Apex	Pointed	Pointed	Blunt	Pointed	Pointed	Pointed	Pointed	Pointed	Blunt	Pointed
Longitudinal Ridges	5	4	X	5	5	5	5	5	5	5
Hydroecium Depth	Medium	Medium	Medium	Deep	Deep	Deep	Medium	Medium	Shallow	Deep
Hydroecium Shape	Claw-shaped	Claw-shaped	Rounded	Rounded	Rounded	Rounded	Rounded	Rounded	Flatted	Rounded
Ostial Teeth	X	X	X	O	O	O	X	X	X	X
Mouthplate	Divided	Divided	Undivided	Undivided	Undivided	Undivided	Divided	Divided	Divided	Divided
Shape of Somatocyst	Fusiform	Oblique Club-shaped	Fusiform	Fusiform	Fusiform	Fusiform	Fusiform	Fusiform	Ovoid	Filiform
Length of Somatocyst Stalk	Short	Long	Short	Short	Short	Short	Short	Short	Short	Short
Somatocyst/Anterior Nectosac	1/2–3/4	1/2	1/2–3/4	1/2	1/2–1/3	1/3–1/4	1/2–1/3	1/2–1/3	1/3	4/5

### Pattern of the protein mass spectra

**Figure 3:**
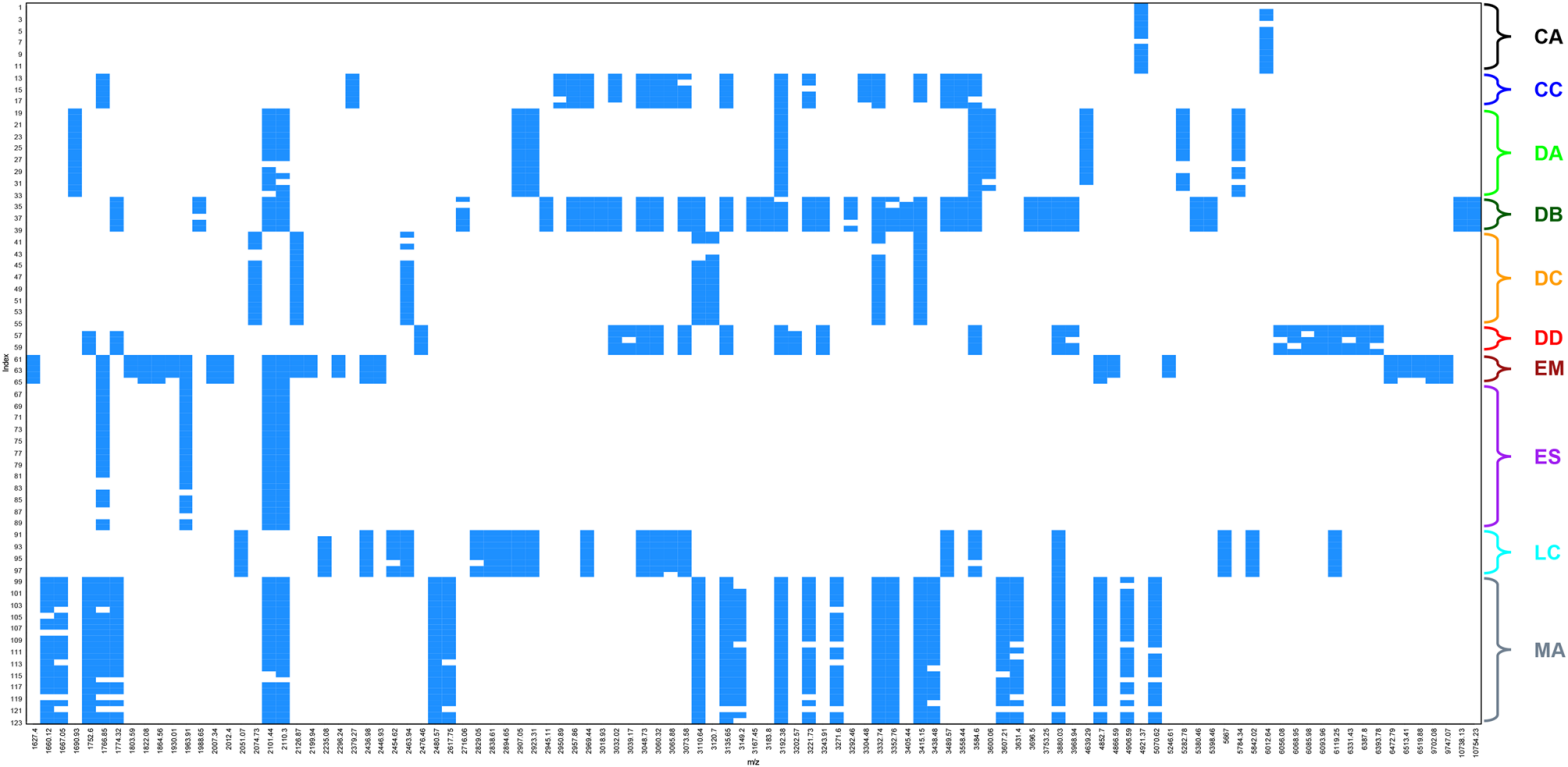
Peak pattern plot of the protein mass spectra of ten Diphyids. X-axis: representative peaks with the highest variation among 123 spectra; Y-axis: number of specimens are displayed in 4 intervals.

Protein mass spectra were obtained from a total of ten species of Diphyidae specimens in a mass peak range between 1.5 and 20 k Dalton (Da) (Table 2). The quality of the mass spectra was controlled separately by eye, and a total of 123 spectra was selected. The same species showed similar peak patterns. On the other hand, noticeable differences in peak patterns were confirmed among the species (Supplementary Fig. S1 online). Figure 3 comprehensively shows the variation in important peaks. Even within the same species, there were slight differences depending on individual. On a larger scale, the peak pattern differed by species. In addition, the peak pattern did not seem to be significantly affected by year of collection, location, and storage period (Supplementary Fig. S2A, B online). Supplementary Fig. S3 online showed the results obtained from Diagonal Discriminant Analysis, which indicated that how strong the most remarkable peaks (protein masses that differed greatly among the species) extracted from the 123 spectra were relatively strong in each species. For instance, *Diphyes chamissonis* Huxley, 1859 had a relatively large amount of 2125–2128 Da compared to the other species. This result confirmed that the level of expression by the peak varied greatly depending on the species. Meanwhile, *Eudoxoides mitra* (Huxley, 1859) and *E. spiralis* showed almost identical features. This was a different result from the overall peak pattern (Fig. 3).

**Table 2:** Collecting information and the number of specimens used in the experiment to obtain DNA sequences and protein mass spectra. * Outgroup.

Species	Abbreviation	Year. month	Cruise	Station	Mass spectra	DNA sequences
*Chelophyes appendiculata*	CA	2018. 08	R/V ISABU	9	6	–
		2019. 10	R/V ISABU	Eddy3	6	1
*Chelophyes contorta*	CC	2020. 05	R/V ISABU	S09	6	3
*Dimophyes arctica*	DA	2019. 10	R/V ISABU	0	15	1
*Diphyes bojani*	DB	2018. 08	R/V ISABU	9	6	2
*Diphyes chamissonis*	DC	2018. 09	R/V DONGBAEK	10	12	1
		2018. 09	R/V DONGBAEK	11	4	4
*Diphyes dispar*	DD	2017. 10	R/V ISABU	11.5	5	3
*Eudoxoides mitra*	EM	2020. 05	R/V ISABU	S12	5	3
*Eudoxoides spiralis*	ES	2020. 05	R/V ISABU	S12	8	–
		2018. 08	R/V ISABU	6	8	–
		2018. 08	R/V ISABU	8	2	1
		2019. 10	R/V ISABU	Eddy1	1	–
		2019. 10	R/V ISABU	Eddy3	6	1
*Lensia cossack*	LC	2019. 10	R/V ISABU	Eddy1	8	1
*Muggiaea atlantica*	MA	2018. 04	R/V DONGBAEK	14	25	3
Hippopodius hippopus*	HH	2019. 10	R/V ISABU	Eddy3	–	1

### Clustering

**Figure 4:**
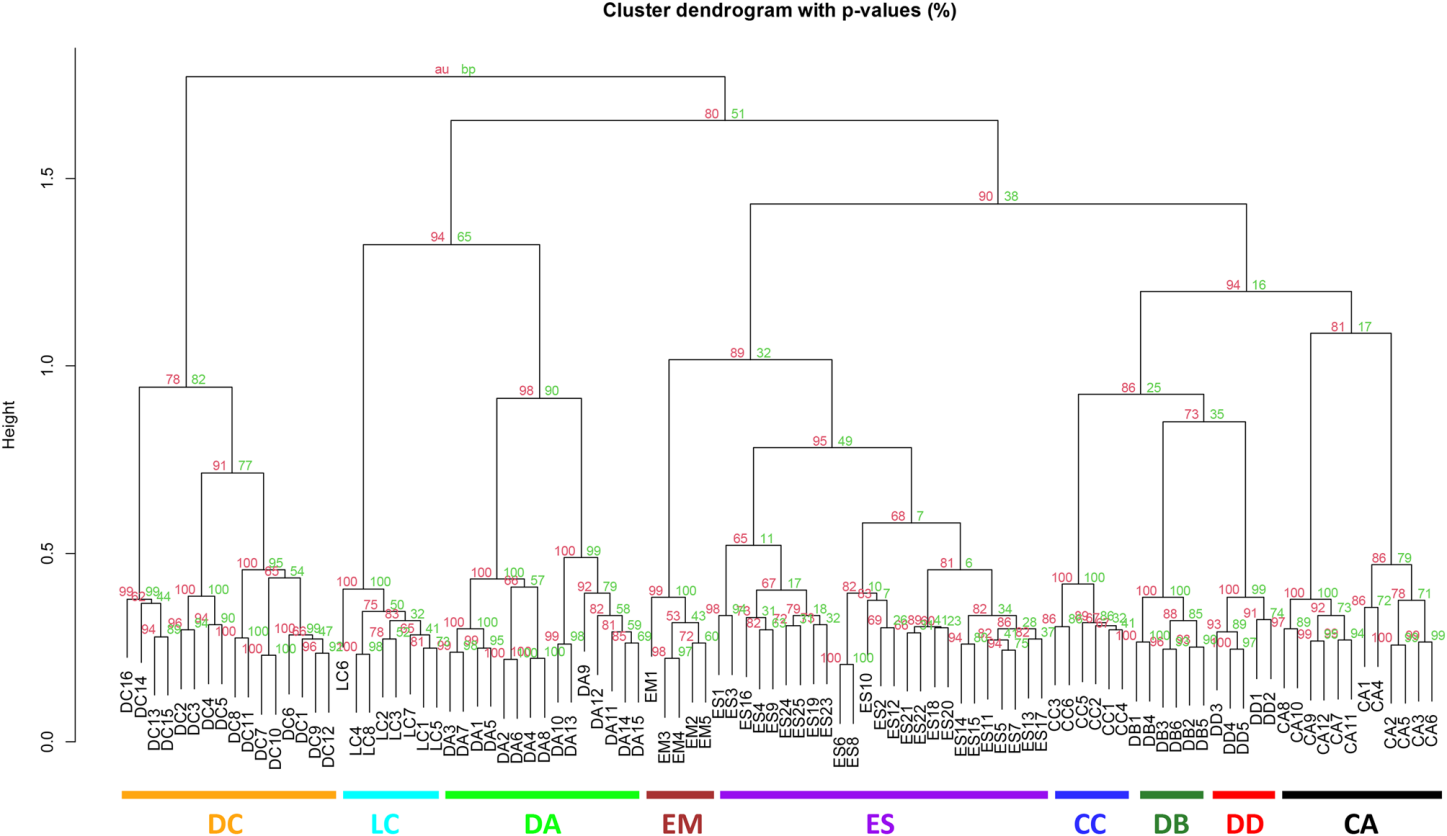
Cluster dendrogram with the *p* values (%) of nine Diphyids (98 spectra). The number of each node dictates the AU (Red) and BP (Green).

Cluster dendrograms with *p* values (%) were obtained with two datasets using a Hellinger-transformed matrix. Dataset 1 included nine species (98 spectra) (Fig. 4) excluding *Muggiaea atlantica* Cunningham, 1892, and Dataset 2 contained ten species (123 spectra) (Supplementary Fig. S4 online) including *M. atlantica*. In Dataset 1, all nine species were divided into individual clusters. Dataset 2 was divided into clusters except for *Chelophyes appendiculata* (Eschscholtz, 1829). The spectra of *M. atlantica* were classified as the outermost cluster. Neither dataset was separated at the genus level, but *E. mitra* and *E. spiralis* clustered closely. These results were consistent with those confirmed in Supplementary Fig. S3 online and were supported by high AU *p* values and rather low BP *p* values. The BP *p* values are at risk of being significantly lower if data are skewed to one side, while the AU *p* values are more reliable because it is more improved and unbiased^15^.

### Non-metric multi-dimensional scaling (NMDS)

**Figure 5:**
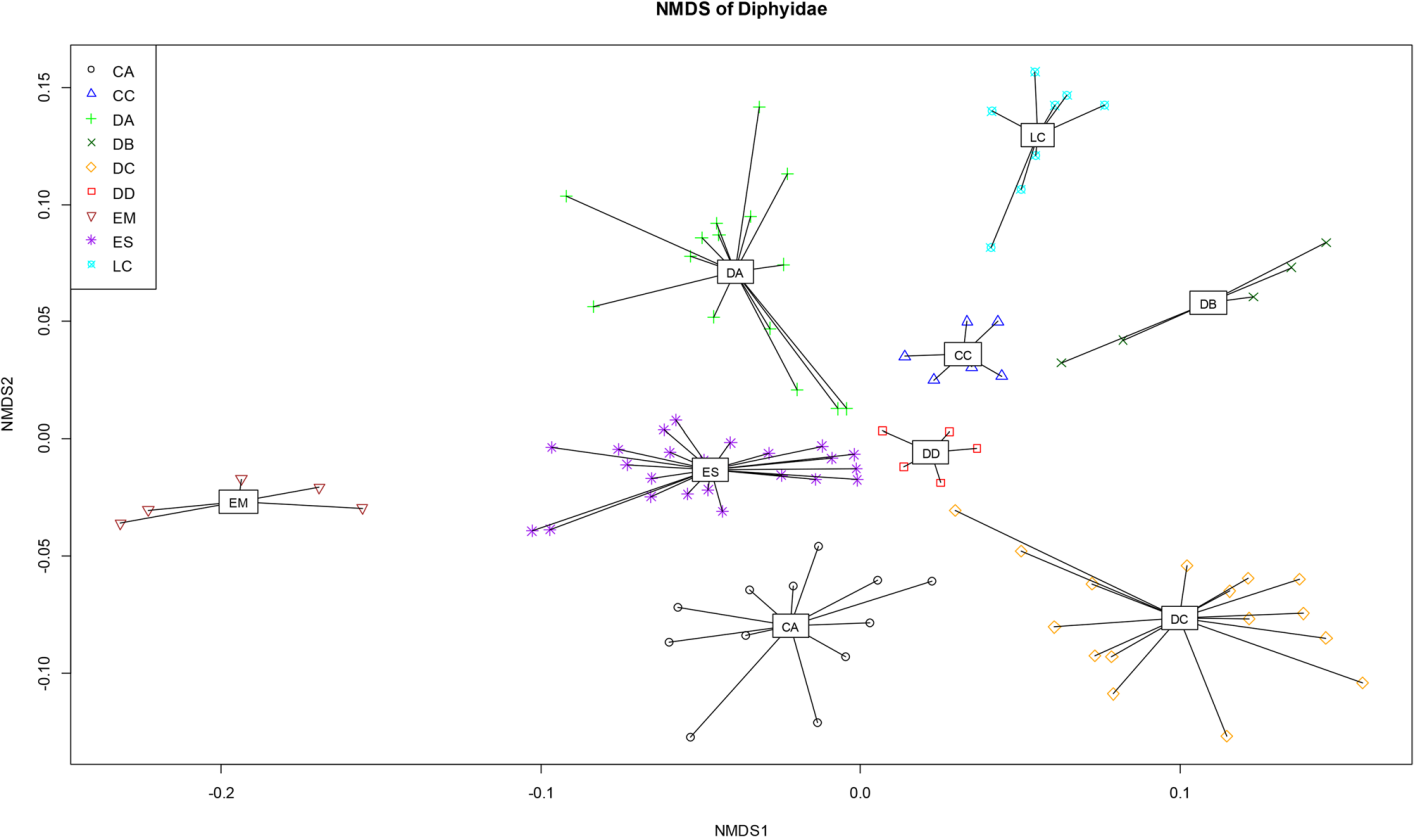
NMDS plot of the protein mass spectra of nine Diphyids from the Hellinger-transformed matrix (98 spectra).

The NMDS analysis was performed based on the Hellinger-transformed matrix of two datasets (Dataset 1-Fig. 5; Dataset 2-Supplementary Fig. S5 online) covered in the Clustering section above. In Dataset 1, the spectra formed groups of the same species regardless of collection year, location, and storage period (homogeneity test: *p* < 0.05, PERMANOVA test: *p* = 0.001). The number of spectra grew respective to the range of the group, but there was no overlap with other species. In Dataset 2, *M. atlantica* was separated clearly, consistent with the clustering results. However, except for *E. mitra, Diphyes bojani* (Eschscholtz, 1825), and *Lensia cossack* Totton, 1941, the remaining six species overlapped (homogeneity test: *p* < 0.05, PERMANOVA test: *p* = 0.001). The stress values shown in Fig. 5 and Supplementary Fig. S5 online were 0.2004 and 0.1706, respectively, which were in a usable range according to Clarke, who suggested that stress values < 0.20 were usable^16^. Considering that our dataset contained 98 and 123 samples of different variables, and since the stress values increased with numbers of samples and variables, the stress values were acceptable.

### Phylogenetic analysis

**Figure 6:**
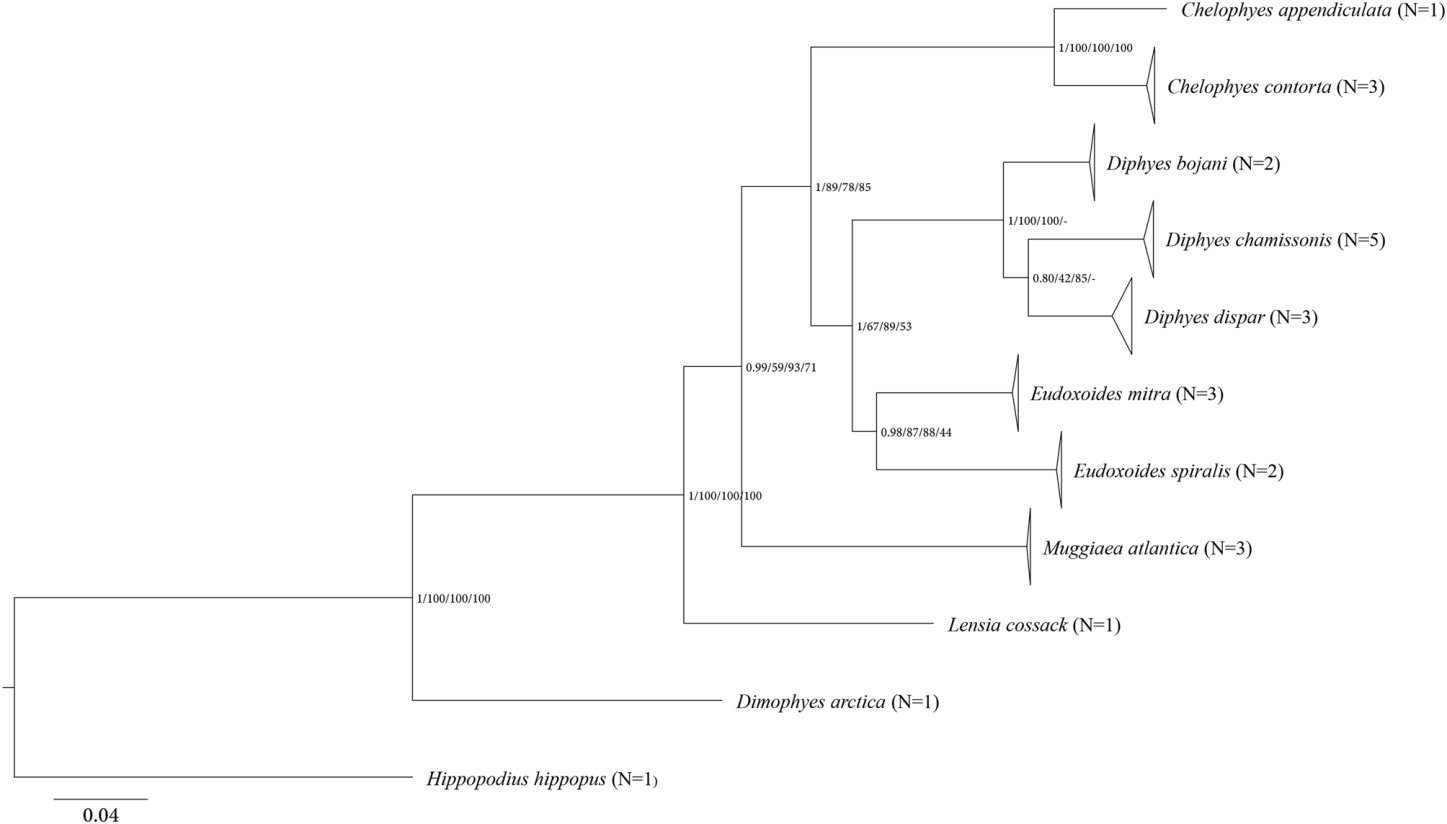
Molecular phylogenetic tree of Diphyidae species based on 25 concatenated sequences. All positions containing gaps and missing data were eliminated. The number of each node dictates the BI/NJ/ML/MP bootstrap values. 'N' indicates the number of each sequence obtained in this study.

A total of 100 new partial sequences for 24 specimens of ten Diphyidae species and a specimen of outgroup species were obtained successfully using four markers (mtCOI, 16S rRNA, 18S rRNA, ITS regions) (Table 2). For five datasets (Concatenated: ~ 4117 bp, mtCOI: ~ 916 bp, 16S rRNA: ~ 691 bp, 18S rRNA: ~ 1803 bp, ITS regions: ~ 797 bp), K2P genetic distances (Supplementary Table S1–5 online) were calculated, and phylogenetic trees were constructed using four algorithms (BI, NJ, ML, and MP). There were differences between the topologies according to phylogenetic tree construction algorithm. In the concatenated tree, the genera of Diphyidae formed each monophylum (Fig. 6) (BI >  = 0.99; NJ >  = 59; ML >  = 67; MP >  = 53). The most distantly related species in Diphyidae was *Dimophyes arctica* (Chun, 1897) (BI = 1; NJ = 100; ML = 100; MP = 100), followed by *L. cossack* (BI = 1; NJ = 100; ML = 100; MP = 100) and *M. atlantica* (BI = 0.99; NJ = 59; ML = 93; MP = 71). The concatenated dataset provided more well-resolved and supported relationships than did the single marker dataset. The topology of the mtCOI-based phylogeny (Supplementary Fig. S6 online) formed was monophyletic by genus, as in the concatenated tree. However, this phylogenetic tree differed from the concatenated tree (Fig. 6) in that *M. atlantica* and *L cossack* formed the monophyletic branch (BI = 0.98; NJ = 62; ML = 60; MP = 37) and the location of *Chelophyes* Totton, 1932 (BI = 1; NJ = 91; ML = 78; MP = 85). The topology of the phylogenetic tree based on 16S rRNA (Supplementary Fig. S7 online) appeared paraphyly in the genus *Eudoxoides* Huxley, 1859. In addition, the topology in *Diphyes* Cuvier, 1817 (BI = 1; NJ = 100; ML = 96; MP = 98/BI = 1; NJ = 95; ML = 90; MP = 87) differed from that of the concatenated tree (Fig. 6). The phylogenetic tree based on 18S rRNA (Supplementary Fig. S8 online) failed to recover species within the genus *Chelophyes, Diphyes,* and *Eudoxoides*. In fact, the distance results for 18S rRNA (Supplementary Table S4 online) showed little difference within 0.005. Nevertheless, as shown in the previous trees, the genera *Dimophyes* Moser, 1925, *Muggiaea* Busch, 1851, and *Lensia* Totton, 1932 were separated (BI = 1; NJ = 100; ML = 100; MP = 98/BI = 0.64; NJ = 52; ML = 66; MP = 57). The phylogenetic tree based on ITS regions (Supplementary Fig. S9 online) showed paraphyly in *Eudoxoides*, like the tree of 16S rRNA. On the other hand, there were differences from the other phylogenetic trees in terms of the locations of *Muggiaea* and *Lensia* (NJ = 79; MP = 92/NJ = 77; MP = 41).

## Discussion

Diphyidae is the only family of the Calycophore Siphonophorans with distinctive bullet-shaped anterior nectophore, making it morphologically unique. However, the species of Diphyidae share many similar morphological features, making it difficult to distinguish species and genera. In fact, the initially described species of Diphyidae were all classified as the genus *Diphyes* and later were split into 45 species of eight genera based on minor differences in features such as ridge number, depth of hydroecium, length of somatocyst, and ostial teeth. Currently, the largest genus within the family is *Lensia*, with 26 species^2^, while the genus *Dimophyes* only had one species recorded. In this study, ten species belonging to six genera of Diphyidae were targeted. One major difficulty with taxonomic analysis of gelatinous Diphyids is that their body consists of > 90% water, and they are immediately dehydrated when fixed with ethanol. This shrivels their body shape, making it difficult to identify key morphological features, increasing the likelihood of misclassification. In a previous Park & Lee study^17^, they used formalin as a fixative for studying *Chelophyes* and *Eudoxoides*, and the formalin-fixed samples were superior in terms of morphology compared to the ethanol-fixed samples in this study (Fig. 1). Table 1 shows the key morphological features that can be observed even after ethanol fixation. Among them, features including hydroecium, ostial teeth, mouth plate, and somatocyst were relatively easy to observe. However, the boundaries of the longitudinal ridges for many species became unclear from folds induced by dehydration, making it difficult to discern species. In fact, *D. arctica* was the only species that was ridge-less with only folds present.

Based on our results, MALDI-ToF MS is a viable alternative to species identification for non-intact gelatinous ethanol-fixed specimens. The ten species targeted in this study showed different spectral patterns (Fig. 3, Supplementary Fig. S1 online), and similar peaks were observed among the same species, regardless of time, sampling location, and duration of storage (Supplementary Fig. S2A, B online, Table 2). These results were consistent with the results of Karger et al.^18^, in which protein mass spectra varied slightly depending on time of collection and sampling location, but not enough to skew species identification. Peak variation between the storage periods of three and 75 days for 70% ethanol-fixed samples was covered by Dvorak et al.^6^, but our study was the first to compare 99% ethanol-fixed samples that had been stored for more than 1 year. In addition, peaks were detected from 4-year-old samples. When MALDI-ToF was attempted with formalin-fixed samples, peaks below 3000 Da resembled those of the matrix only. In comparison to the ethanol-fixed *C. appendiculata*, the strong peak observed at 4920 Da also was observed in the formalin-fixed spectra but with considerably less intensity, rendering it unusable (Supplementary Fig. S2C, D online). This is believed to be due to the cross-linking nature of the formalin-induced proteins^19^.

The Hellinger-transformed matrix was used for visualization, as per recommendation by Rossel & Martinez Arbizu^20^ as the best transformation method for species identification. With the exception of *M. atlantica*, the dataset of the other nine species showed valid discrimination in both cluster analysis and NMDS (Fig. 4, 5). However, without prior morphological species identification, *C. appendiculata* from Fig. 4 could have been divided into two species. The two groups of *C. appendiculata* differed in sampling time and location. The peak pattern (Fig. 3) and raw spectra (Supplementary Fig. S2A, B online) were similar (especially 4925 Da), nonetheless, and even formed a single group in the NMDS plot (Fig. 5). The difference in detailed peaks appeared to have been greatly overestimated, causing the division into two groups. However, in agreement with aforementioned by Karger et al.^18^, the difference seemed minimal to skew or influence species identification. Granted, it is necessary to obtain and compare more samples with varying conditions (peaks, timing, etc.) to fully understand these variations.

No notable associations between the morphological features mentioned in Table 1 and the clusters (Figs. 4), but Fig. 4 showed that the blunt apex species (*D. arctica* and *L. cossack*) were bound to the same cluster. It was evident from cluster analysis and NMDS of the dataset containing ten species (Supplementary Fig. S4, 5 online) that *M. atlantica* was skewed to one side. The NMDS plot of the other species overlapped with one another, rendering them indistinguishable. This seemed likely due to the more accentuated *M. atlantica* showed peaks than those of the rest of the species, resulting in a Hellinger-transformed matrix more centralized toward *M. atlantica*. The peak pattern (Fig. 3) and top-ranking feature (Supplementary Fig. S3 online) showed significantly different peak expressions per species. Peaks shared by many species tended to have decreased t-scores of approximately − 10, and peaks rarely found in other species, such as 3414–3416, 1752–1753, and 3331–3332 Da, showed high levels of expression. Supplementary Fig. S4, 5 online grouped the dataset of ten species with a large peak difference, resulting in the graph skewing to one side and resembling that of a phylogenetic tree with an incorrect outgroup. Therefore, it would be more advantageous to compare species by species for higher accuracy. Similar results were seen with DNA analysis. Our results showed that *M. atlantica* differed from the other species by 0.270–0.465 (mtCOI) and 0.257–0.432 (16S rRNA) (Supplementary Table S2, 3 online). According to Zheng et al.^21^, the intra-family mtCOI and 16S rRNA K2P distance of hydrozoan ranged from 0.104 to 0.248 (average = 0.192) and 0.073–0.287 (average = 0.198), respectively. In this context, the difference between *M. atlantica* and other species was beyond the family level. Furthermore, phylogenetic analysis showed that *M. atlantica* was the third-most distant in terms of relationship within Diphyidae (Fig. 6; Supplementary Fig. S6–8 online). It is plausible that the difference in genetic distance may have affected protein expression and pattern differences. However, *D. arctica* was far more genetically distant than *M. atlantica*, but the protein patterns did not reflect this. Based on this result, it cannot be said that protein peak patterns directly reflect phylogenetic relationship. In fact, clustering results were not separated at the genus level with the exception of *Eudoxoides* nor were the topologies of the cluster analysis and phylogeny tree consistent with one another because the MALDI-ToF MS method does not account for composition or molecular function of proteins, but instead simply compares the pattern of the protein peaks^22^. Granted, as mentioned earlier, a difference in genetic distance can be reflected in protein expression, just not in proportion to genetic distance.

Peter et al. showed mtCOI NJ trees including six (*D. bojani*, *D. chamissonis*, *D. dispar*, *E. mitra*, *E. spiralis*, *M. atlantica*) of our target species^23^. The same topologies were supported in the phylogenetic trees of this study, further extending phylogenetic locations of four species (*C. appendiculata*, *Chelophyes contorta* (Lens & van Riemsdijk, 1908), *D. arctica*, *L. cossack*) based on a total of four gene concatenated sequences using four algorithms (BI, NJ, ML, MP) (Fig. 6; Supplementary Fig. S6–99 online). Each dataset consisted of the same specimens, all of which formed a monophyletic group with clear discrimination. However, depending on the marker and algorithm used, the topology at the genus level differed. In addition, the phylogenetic tree produced with 18S rRNA (Supplementary Fig. S8 online) could not distinguish the phylogenetic position of the genera *Chelophyes*, *Diphyes*, and *Eudoxoides*. This result was consistent with the results of a previous study^24^, which suggested that the 18S rRNA marker was not useful in species differentiation at the genus or species level.

The association between the phylogenetic tree and morphological features was noted in genus-specific characteristics (Claw-shaped hydroecium in *Chelophyes*; Ostial teeth in *Diphyes*). Species with claw-shaped hydroecium and ostial teeth characteristics were monophyletic, respectively. It also confirmed that *D. arctica*, without longitudinal ridges, and another nine species were divided distinctly and phylogenetically. In concatenated and 16S rRNA (Fig. 6, Supplementary Fig. S7 online), eight species that had a pointed apex were established as the monophyletic branch, and the blunt apex species, *D. arctica* and *L. cossack*, were separated. This was similar to what appeared in Fig. 4 (the aforementioned binding of *D. arctica* and *L. cossack*). Furthermore, the results of the phylogenetic analysis suggested that *D. arctica* and *L. cossack* were located at the outermost location within Diphyidae, close to the nearest ancestor. Taken comprehensively, these results indicated that the ancestral Diphyidae initially had neither ridges nor an apex and subsequently evolved to become more angular, with ridges forming, and apex becoming pointed. Indeed, Abylidae L. Agassiz, 1862, Siphonophorans, known to have many angles, were of evolutionary descendant and were located in the innermost phylogenetic position of Diphyidae^25^. However, at present this conviction is incomplete, and we need to obtain more information about a wider variety of species.

We have for the first time confirmed that MALDI-ToF can be a valid method of species identification for the morphologically “difficult” group Diphyidae. This method used in conjunction with morphological analysis and DNA information helped to ensure efficient and accurate species identification. There are still some limitations of using this method alone. First, MALDI-ToF analysis cannot yield any insights regarding phylogenetic relationships. It is still noteworthy that E*. mitra* and *E. spiralis* were clustered closely and showed similar peak expressions in the top ranks despite significant differences in peak pattern (Supplementary Fig. S1, 3 online). We suspected that there might be differences between the representative peaks selected from matrices and the real peaks. Based on this finding, it was expected that the absence of phylogenetic analysis could be supplemented by genus-specific peaks. Second, the lack of existing data on Diphyidae spectra limited the methodology to application as a supplement to morphological and DNA analyses. In comparison to DNA barcoding, MALDI-ToF MS is cheaper and less time consuming, with a higher sample throughput. Therefore, it is expected that fast accumulation of data will be possible. For this to be possible, it will be necessary to establish a shared database system among researchers, such as the NCBI. Finally, organisms, such as Diphyidae, which are polymorphic by generational rotation or colony formation, might differ in protein expression depending on life stage. To address this, researchers in future studies will have to focus on profiling data for all stages of a species based on spectra of both posterior nectophore and eudoxids.

## Conclusions

For the first time, we applied a fast and accurate using MALDI-ToF MS method for identification of ten species of Diphyidae that are morphologically difficult to discriminate, and assessed the potential for use. Protein mass spectra showed clear differences between species and confirmed the validity of the identification tool. Ethanol-but not formalin-fixed samples should be used, and old ethanol-fixed samples can also be used because they are not affected by storage period. This novel method has breakthroughs for cost and time. In addition, the morphological characteristics and partial sequences of four genes (mtCOI, 16S rRNA, 18S rRNA, ITS regions) were obtained, and the associations were compared to each other. The tools and reference data established in this study can be used as a fundamental source for multilateral understanding of Siphonophore.

## Methods

### Sample collection

The samples used in this study were collected in 2017–2020, during a, R/V ISABU cruise in the Northwest Pacific Ocean area affected by the Kuroshio Current, and in 2018, during an R/V DONGBAEK cruise in the Jeju and along the southern coast of Korea (Fig. 1). On the R/V ISABU, a Multiple Opening/Closing Net and Environmental Sensing System (MOCNESS, 1 × 1 m^2^, mesh size: 200 µm) was towed between a depth of 200 m and the surface. On the R/V DONGBAEK, we towed a plankton net (mesh size: 200 µm, Ø: 60 cm) vertically from the bottom to the surface. The mouth of the net was equipped with a flowmeter (Hydro-Bios, Germany) to determine the volume of filtered water during each tow. Immediately after towing, we split the samples into 1/2 aliquots using a Folsom plankton splitter. Each aliquot was fixed with neutralized 5% formalin solution or 99% ethanol. In this study, we only used ethanol-fixed samples.

### Morphological analysis

Siphonophore specimens were sorted from 99% ethanol samples using Live Insect Forceps (26029-10, Fine Science Tools Inc., Foster City, CA, USA) under a stereomicroscope (Olympus SZX7, Tokyo, Japan). We identified specimens using descriptions, illustrations, and terminology from the literature^2,26–29^. For morphological records, digital photographs of specimens were produced at various focal points using a digital camera (Olympus PEN Lite E-PL3, Tokyo, Japan) connected to the stereomicroscope with side lights on the dark field. The obtained multi-focus photos were stacked using Helicon Focus 7 software (Version: 7.5.1), Afterward, only the object was cropped and moved to a black background using Adobe Photoshop CS6 software (Version: 13.0).

### MADLI-ToF MS

Part of the gelatinous substance of each individual separated with Live Insect Forceps or the entire individual were put into a 1.5 ml tube to prepare for protein mass measurement. After evaporating the ethanol, the samples were dried in a vacuum dryer for 30 min. 10–20 μl of α-Cyano-4-hydroxy-cinnamic acid (HCCA) matrix (Acetonitrile 50%; Ultra-pure water 47.5%; Trifluoroacetic acid 2.5%; supersaturated HCCA (30 mg for a total of 1 ml of matrix)) was added to each tube and incubated at room temperature for at least 30 min. Matrix-specimen solutions were centrifuged at 12,000 rpm for 1 min. 2 μl of the supernatant was loaded onto the target plate of the MALDI-ToF MS equipment (AXIMA Confidence MALDI ToF-Mass Spectrometer; Shimadzu), and the solutions were air-dried completely to crystalize them. Protein mass spectra were measured in the range of 1–20 k Dalton on MALDI-MS Application Launchpad 2.9.2 (Shimadzu Biotech) software using positive-ion linear mode with a laser power of 80. For each loading spot, 100 profiles were repeated ten times and summed into one spectrum. Each protein mass spectrum was exported in ASCII format and imported to Data Explorer version 4.5 software for range trimming to 1.5–20 kDa.

### Protein mass spectra analysis

Data processing and analysis of protein mass spectra were conducted using MALDIquantForeign (ver. 0.12)^30^, MALDIquant (ver. 1.19.3)^31^, and MALDIrppa (ver. 1.0.5)^32^ packages in the RStudio program (version: 1.4.1103, RStudio Inc., Boston, MA, USA; R version: 4.0.5). The workflow in R referred to manual^33^ and literature^22^. Spectra were proceeded square root transforming and smoothing (Savitzky-Golay method), baseline removing (SNIP baseline estimation method), normalizing (Total-Ion-Current-calibration (TIC) method) implemented in MALDIquant. Significant peaks were detected with a signal-to-noise ratio (SNR) of 6 and a half window size of 7. Peaks were binned repeatedly with the “binpeaks” command from MALDIquant, with a tolerance of 0.001 and a minimum frequency of 0.05. The feature matrix obtained through this process was Hellinger-transformed and visualized through cluster analysis, diagonal discriminant analysis (DDA), and non-metric multi-dimensional scaling (NMDS) analysis.

The cluster dendrogram was established with the pvclust (ver. 2.2) R package^15^ using Ward’s 2D clustering algorithm with Euclidean distances and 10,000 bootstrapping replications. The approximately unbiased (AU) *p* values and bootstrap probability (BP) values were calculated in the dendrogram. DDA was performed with the sda^34^ function to find the peaks with the highest variation among the Diphyidae species and to calculate the t-score for feature ranking. An NMDS plot was created with the vegan (ver. 2.5.6) R package^35^ based on the Bray–Curtis Dissimilarity distance with k = 2. ANOVA was conducted utilizing the betadisper function provided with the vegan package to test the multivariate homogeneity of group dispersion. PERMANOVA was performed using the Adonis tool to test the fit of the data with 999 permutations. In addition, a plot using the ‘peakPatterns’ function of MALDIrppa (ver. 1.0.5) was obtained and simplified to a minimum frequency of 0.8.

### DNA extraction, amplification, and sequencing

For DNA extraction, the remaining parts of the individuals used to measure the protein mass were transferred to ultra-pure water for three hours to wash and remove the ethanol. A LaboPass™ Genomic Isolation-Tissue miniprep kit (Cosmogenetech Co., Seoul, Korea) was used to extract genomic DNA following the manufacturer’s protocols. We amplified four genetic markers: mitochondrial cytochrome c oxidase subunit I (mtCOI); 16S ribosomal RNA (16S rRNA); 18S ribosomal RNA (18S rRNA); and Internal transcribed spacer regions (ITS regions) via polymerase chain reaction (PCR) using the AccuPower® PCR PreMix (Bioneer Co., Daejeon, Korea), and performed thermal cycling using a TaKaRa Thermal Cycler Dice Touch TP350 (Takara Bio Inc., Kusatsu, Japan). PCR products consisted of 5 μl premix, 15 μl ultra-pure water, 3 μl DNA template, and 1 μl each of the forward and reverse primers to achieve a 25 μl total reaction volume per tube.

The MtCOI genes (about 700–800 bp) were amplified using modified jgLCO1490 & jgHCO2198 primers with thermo-cycling conditions: initial 5 min at 95 °C, followed by 42 cycles of 20 s at 95 °C, 1 min at 48 °C, and 1 min at 72 °C, and ending with a final 5 min at 72 °C referenced and modified from Geller et al.^36^. COF & COR primers were used with the following thermo-cycling conditions: 5 cycles of 50 s at 94 °C, 50 s at 45 °C, and 2 min at 70 °C, followed by 30 cycles of 50 s at 94 °C, 50 s at 50 °C, and 2 min at 68°C^37^. 16S rRNA (about 600 bp) genes were amplified using SHA & SHB primers with the following thermo-cycling conditions: 30 cycles of 20 s at 94 °C, 45 s at 50 °C, and 2 min at 68°C^37^. 18S rRNA (about 1800 bp) genes were amplified using EukA & EukB primers with the following thermo-cycling conditions: 30 cycles of 10 secs at 94 °C, 1 min at 38 °C, and 3 min at 72 °C, and a final 2 min at 94°C^38,39^. ITS regions (about 750 bp) genes were amplified using IFS & IRS primers with the following thermo-cycling conditions: 24 cycles of 20 secs at 94 °C, 45 s at 51 °C, and 1 min 30 s at 72°C^37^.

Amplifications were confirmed by electrophoresis on a 1% agarose gel (AGAROSE I™, Amresco Inc., Solon, OH, USA; LaboPass™ Buffer 50X TAE, Cosmogenetech Co., Seoul, Korea) with Staining STAR (Dynebio Inc., Seongnam, Korea) for 20 min at 100 V with a 100 bp DNA ladder (Bioneer Co., Daejeon, Korea). PCR products were purified using a LaboPass™ PCR Purification Kit (Cosmogenetech Co., Seoul, Korea) following the manufacturer’s protocols. Purified PCR products were sent to Bionics Inc. (Seoul, Korea) for DNA sequencing. For sequencing, an ABI automatic capillary sequencer was used with the same set of primers as used for amplification. Due to its long length, the internal primer also was used for the 18S rRNA sequencing^40^. All obtained sequences were visualized using Finch TV software (ver. 1.4.0) (https://digitalworldbiology.com/FinchTV; Geospiza Inc., USA). The quality of each sequence was evaluated, and low-resolution peaks were checked by comparing forward and reverse strands. BLAST^41^ search confirmed the obtained sequences as Siphonophores without contaminants. Sequence information from this study was deposited in the GenBank database (MZ230437–MZ230486, MZ230526–MZ230550, MZ292030, MZ292031, MZ292870–MZ292892).

### Phylogenetic analysis

Each dataset consisted of sequences of 24 specimens of ten Diphyids species. We used a specimen of *Hippopodius hippopus* (Forsskål, 1776) belonging to the family Hippopodiidae Kölliker, 1853, as an outgroup. Sequences were aligned using the ClustalW algorithm^42^ with default parameters embedded in Molecular Evolutionary Genetics Analysis 7 software (MEGA7, Version: 7.0.26)^43^. The genetic distances between alignment sequences were calculated using the Kimura 2-parameter (K2P) model^44^ with complete deletion by MEGA7 software.

Phylogenetic analyses were performed using the Neighbor-Joining (NJ), Maximum Parsimony (MP), Maximum Likelihood (ML), and Bayesian Inference (BI) approaches. The best-fit evolutionary model for phylogenetic analysis was calculated using jModelTest software (Version: 2.1.7)^45,46^ with the Akaike information criterion (AIC)^47–49^ (Concatenated: GTR + I + G; mtCOI: GTR + I + G; 16S rRNA: TVM + I + G; 18S rRNA: TIM2 + I; ITS regions: TIM2 + I + G). Gap calibration was conducted using FastGap (Version: 1.2)^50,51^. NJ, MP, and ML analyses were performed using MEGA7 software, PAUP4 software^52^, and IQTree web server^53^, respectively, with 1000 bootstrapping replicates for phylogenetic tree reconstruction^54^. The BI tree was constructed using MrBayes software (Version: 3.2.6)^55–57^ based on the following model parameters. Markov Chain Monte Carlo (MCMC) was run with the following parameters: ngen = 1,000,000, nchains = 4, samplefreq = 100, savebrlens = yes, and printfreq = 1000. The BI trees were constructed using the “sump” command with burin = 250 to summarize the parameters and the “sumt” command with burin = 250 to summarize the tree. All trees were visualized using FigTree (version: 1.4.2). The concatenated dataset of ML was partitioned and applied to the model according to each marker. For NJ, MP, and ML, no separate partitioning was performed on the concatenated dataset.

## Supplementary Information

Supplementary Information 1.

Supplementary Information 2.

Supplementary Information 3.

Supplementary Information 4.

Supplementary Information 5.

Supplementary Information 6.

Supplementary Information 7.

Supplementary Information 8.

Supplementary Information 9.

Supplementary Information 10.

Supplementary Information 11.

Supplementary Information 12.

Supplementary Information 13.

Supplementary Information 14.

## Data Availability

The datasets generated and/or analyzed during the current study are available in Genbank (https://www.ncbi.nlm.nih.gov/genbank/) and this published article (and its supplementary materials).

## References

[1] Pugh P (1974). The vertical distribution of the siphonophores collected during the SOND cruise, 1965. J. Mar. Biol. Assoc. U.K..

[2] Grossmann MM, Collins AG, Lindsay DJ (2014). Description of the eudoxid stages of *Lensia havock* and *Lensia leloupi* (Cnidaria: Siphonophora: Calycophorae), with a review of all known *Lensia* eudoxid bracts. Syst. Biodivers..

[3] Dunn CW, Wagner GP (2006). The evolution of colony-level development in the Siphonophora (Cnidaria: Hydrozoa). Dev. Genes. Evol..

[4] Karas M, Hillenkamp F (1988). Laser desorption ionization of proteins with molecular masses exceeding 10,000 daltons. Anal. Chem..

[5] Clark AE, Kaleta EJ, Arora A, Wolk DM (2013). Matrix-assisted laser desorption ionization–time of flight mass spectrometry: a fundamental shift in the routine practice of clinical microbiology. Clin. Microbiol. Rev..

[6] Dvorak V (2014). Identification of phlebotomine sand flies (Diptera: Psychodidae) by matrix-assisted laser desorption/ionization time of flight mass spectrometry. Parasit. Vectors.

[7] Rossel S, Arbizu PM (2019). Revealing higher than expected diversity of Harpacticoida (Crustacea: Copepoda) in the North Sea using MALDI-TOF MS and molecular barcoding. Sci. Rep..

[8] Feltens R, Görner R, Kalkhof S, Gröger-Arndt H, von Bergen M (2010). Discrimination of different species from the genus *Drosophila* by intact protein profiling using matrix-assisted laser desorption ionization mass spectrometry. BMC Evol. Biol..

[9] Mazzeo MF (2008). Fish authentication by MALDI-TOF mass spectrometry. J. Agric. Food Chem..

[10] Kaufmann C, Schaffner F, Ziegler D, Pflueger V, Mathis A (2012). Identification of field-caught *Culicoides* biting midges using matrix-assisted laser desorption/ionization time of flight mass spectrometry. Parasitology.

[11] Laakmann S (2013). Comparison of molecular species identification for North Sea calanoid copepods (Crustacea) using proteome fingerprints and DNA sequences. Mol. Ecol. Resour..

[12] Lassen S, Wiebring A, Helmholz H, Ruhnau C, Prange A (2012). Isolation of a Nav channel blocking polypeptide from *Cyanea capillata* medusae–a neurotoxin contained in fishing tentacle isorhizas. Toxicon.

[13] Lazcano-Pérez F, Arellano RO, Garay E, Arreguín-Espinosa R, Sánchez-Rodríguez J (2017). Electrophysiological activity of a neurotoxic fraction from the venom of box jellyfish *Carybdea marsupialis*. Comp. Biochem. Physiol. C: Toxicol. Pharmacol..

[14] Helmholz H, Naatz S, Lassen S, Prange A (2008). Isolation of a cytotoxic glycoprotein from the Scyphozoa *Cyanea lamarckii* by lectin-affinity chromatography and characterization of molecule interactions by surface plasmon resonance. J. Chromatogr. B.

[15] Suzuki R, Shimodaira H (2006). Pvclust: an R package for assessing the uncertainty in hierarchical clustering. Bioinformatics.

[16] Clarke KR (1993). Non-parametric multivariate analyses of changes in community structure. Aust. J. Ecol..

[17] Park N, Lee W (2020). Four new records of family Diphyidae (Hydrozoa: Siphonophorae) in Korean waters. J. Spec. Res..

[18] Karger A, Bettin B, Gethmann JM, Klaus C (2019). Whole animal matrix-assisted laser desorption/ionization time-of-flight (MALDI-TOF) mass spectrometry of ticks–are spectra of *Ixodes ricinus* nymphs influenced by environmental, spatial, and temporal factors?. PLoS ONE.

[19] Tayri-Wilk T (2020). Mass spectrometry reveals the chemistry of formaldehyde cross-linking in structured proteins. Nat. Commun..

[20] Rossel S, Martínez Arbizu P (2018). Effects of sample fixation on specimen identification in biodiversity assemblies based on proteomic data (MALDI-TOF). Fron. Mar. Sci..

[21] Zheng L, He J, Lin Y, Cao W, Zhang W (2014). 16S rRNA is a better choice than COI for DNA barcoding hydrozoans in the coastal waters of China. Acta Oceanol. Sin..

[22] Yeom J, Park N, Jeong R, Lee W (2021). Integrative description of cryptic *Tigriopus* species from Korea using MALDI-TOF MS and DNA barcoding. Front. Mar. Sci..

[23] Peter S (2016). Molecular characterization and phylogenetic analysis of *Diphyes dispar* (Siphonophora: Diphyidae) from the Laccadive Sea, off the south-west coast of Arabian Sea, Indian Ocean. Int. J. Fish Aquat. Stud..

[24] Dunn CW, Pugh PR, Haddock SH (2005). Molecular phylogenetics of the Siphonophora (Cnidaria), with implications for the evolution of functional specialization. Syst. Biol..

[25] Munro C (2018). Improved phylogenetic resolution within Siphonophora (Cnidaria) with implications for trait evolution. Mol. Phylogenet. Evol..

[26] Totton AK (1954). Siphonophora of the Indian Ocean together with systematic and biological notes on related specimens from other oceans. Disc Rep.

[27] Totton, A. K., Bargmann, H. E. & British Museum (Natural History). *A synopsis of the Siphonophora*. (British Museum (Natural History), 1965).

[28] Mapstone, G. M. *Siphonophora (Cnidaria, Hydrozoa) of Canadian Pacific waters*. (NRC Research Press, 2009).

[29] Nishiyama EY, Araujo EM, Oliveira OM (2016). Species of *Lensia* (Cnidaria: Hydrozoa: Siphonophorae) from southeastern Brazilian waters. Zoologia (Curitiba).

[30] MALDIquantForeign: Import/Export routines for MALDIquant (2015).

[31] Gibb S, Strimmer K (2012). MALDIquant: a versatile R package for the analysis of mass spectrometry data. Bioinformatics.

[32] Palarea-Albaladejo J, Mclean K, Wright F, Smith DG (2018). MALDIrppa: quality control and robust analysis for mass spectrometry data. Bioinformatics.

[33] Gibb, S. & Strimmer, K. *Species Identification using MALDIquant manual*. http://www.strimmerlab.org/software/maldiquant/. (2015).

[34] Ahdesmäki M, Strimmer K (2010). Feature selection in omics prediction problems using cat scores and false nondiscovery rate control. Ann. Appl. Stat..

[35] vegan: Community Ecology Package. R package version 2.5-2. 2018 (2018).

[36] Geller J, Meyer C, Parker M, Hawk H (2013). Redesign of PCR primers for mitochondrial cytochrome c oxidase subunit I for marine invertebrates and application in all-taxa biotic surveys. Mol. Ecol. Resour..

[37] Schuchert P (2018). DNA barcoding of some Pandeidae species (Cnidaria, Hydrozoa, Anthoathecata). Rev. Suisse Zool..

[38] Medlin L, Elwood HJ, Stickel S, Sogin ML (1988). The characterization of enzymatically amplified eukaryotic 16S-like rRNA-coding regions. Gene.

[39] Collins, A. G. Towards understanding the phylogenetic history of Hydrozoa: hypothesis testing with 18S gene sequence data. *Scientia Marina* (2000).

[40] Strychar KB, Hamilton LC, Kenchington EL, Scott DB (2005). Cold-water Corals and Ecosystems.

[41] Altschul SF, Gish W, Miller W, Myers EW, Lipman DJ (1990). Basic local alignment search tool. J. Mol. Biol..

[42] Thompson JD, Higgins DG, Gibson TJ (1994). CLUSTAL W: improving the sensitivity of progressive multiple sequence alignment through sequence weighting, position-specific gap penalties and weight matrix choice. Nucleic Acids Res..

[43] Kumar S, Stecher G, Tamura K (2016). MEGA7: molecular evolutionary genetics analysis version 7.0 for bigger datasets. Mol. Biol. Evol..

[44] Kimura M (1980). A simple method for estimating evolutionary rates of base substitutions through comparative studies of nucleotide sequences. J. Mol. Evol..

[45] Darriba D, Taboada GL, Doallo R, Posada D (2012). jModelTest 2: more models, new heuristics and parallel computing. Nat. Methods.

[46] Guindon S, Gascuel O (2003). A simple, fast and accurate method to estimate large phylogenies by maximum-likelihood. Syst. Biol..

[47] Akaike H (1974). A new look at the statistical model identification. IEEE Trans. Autom. Control.

[48] Yamaoka K, Nakagawa T, Uno T (1978). Application of Akaike's information criterion (AIC) in the evaluation of linear pharmacokinetic equations. J. Pharmacokinet. Biopharm..

[49] Hurvich CM, Tsai C-L (1989). Regression and time series model selection in small samples. Biometrika.

[50] Simmons MP, Ochoterena H (2000). Gaps as characters in sequence-based phylogenetic analyses. Syst. Biol..

[51] Young ND, Healy J (2003). GapCoder automates the use of indel characters in phylogenetic analysis. BMC Bioinform..

[52] Swofford DL, Sullivan J (2003). Phylogeny inference based on parsimony and other methods using PAUP*. Phylogenet. Handb. Pract. Approach DNA and Protein Phylogeny.

[53] Trifinopoulos J, Nguyen L-T, von Haeseler A, Minh BQ (2016). W-IQ-TREE: a fast online phylogenetic tool for maximum likelihood analysis. Nucleic Acids Res..

[54] Felsenstein J (1985). Confidence limits on phylogenies: an approach using the bootstrap. Evolution.

[55] Huelsenbeck JP, Ronquist F (2001). MRBAYES: Bayesian inference of phylogenetic trees. Bioinformatics.

[56] Ronquist F, Huelsenbeck JP (2003). MrBayes 3: Bayesian phylogenetic inference under mixed models. Bioinformatics.

[57] Ronquist F (2012). MrBayes 3.2: efficient Bayesian phylogenetic inference and model choice across a large model space. Syst. Biol..

