# Supplementary material for: Novel attempt at discrimination of a bullet-shaped siphonophore (Family Diphyidae) using matrix-assisted laser desorption/ionization time of flight mass spectrometry (MALDI-ToF MS)
Source: Sci Rep. 2021 Sep 24;11:19077. doi: 10.1038/s41598-021-98724-z (PMC8463557; doi:10.1038/s41598-021-98724-z)
Supplement: Supplementary file 1 — Supplementary Information 1. [file 41598_2021_98724_MOESM1_ESM.pdf]

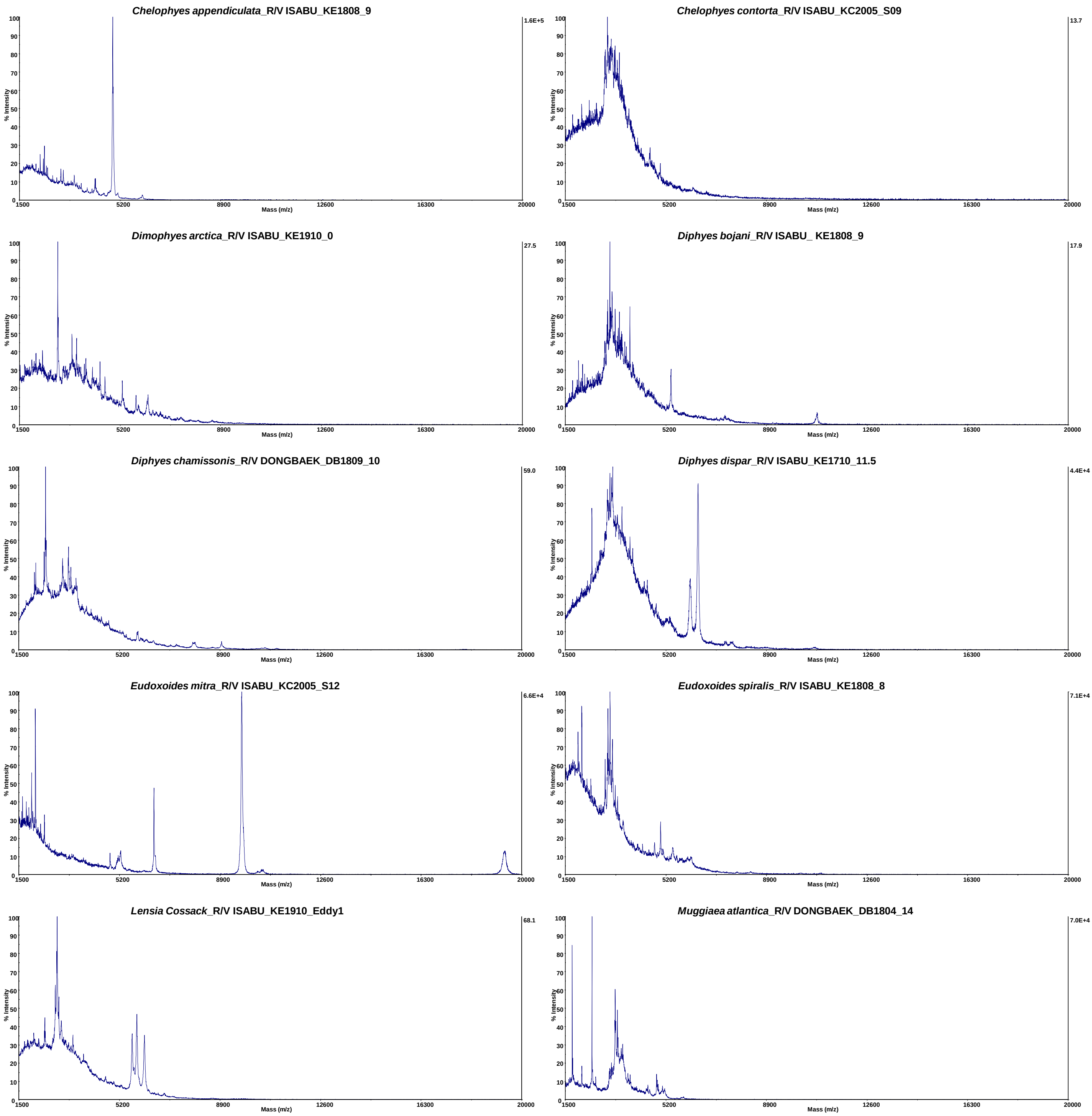

Figure S1. Raw protein mass spectra of the ten Diphyids. X-axis: protein mass (m/z); Y-axis: intensity of protein expression.
