# Supplementary material for: Novel attempt at discrimination of a bullet-shaped siphonophore (Family Diphyidae) using matrix-assisted laser desorption/ionization time of flight mass spectrometry (MALDI-ToF MS)
Source: Sci Rep. 2021 Sep 24;11:19077. doi: 10.1038/s41598-021-98724-z (PMC8463557; doi:10.1038/s41598-021-98724-z)
Supplement: Supplementary file 2 — Supplementary Information 2. [file 41598_2021_98724_MOESM2_ESM.pdf]

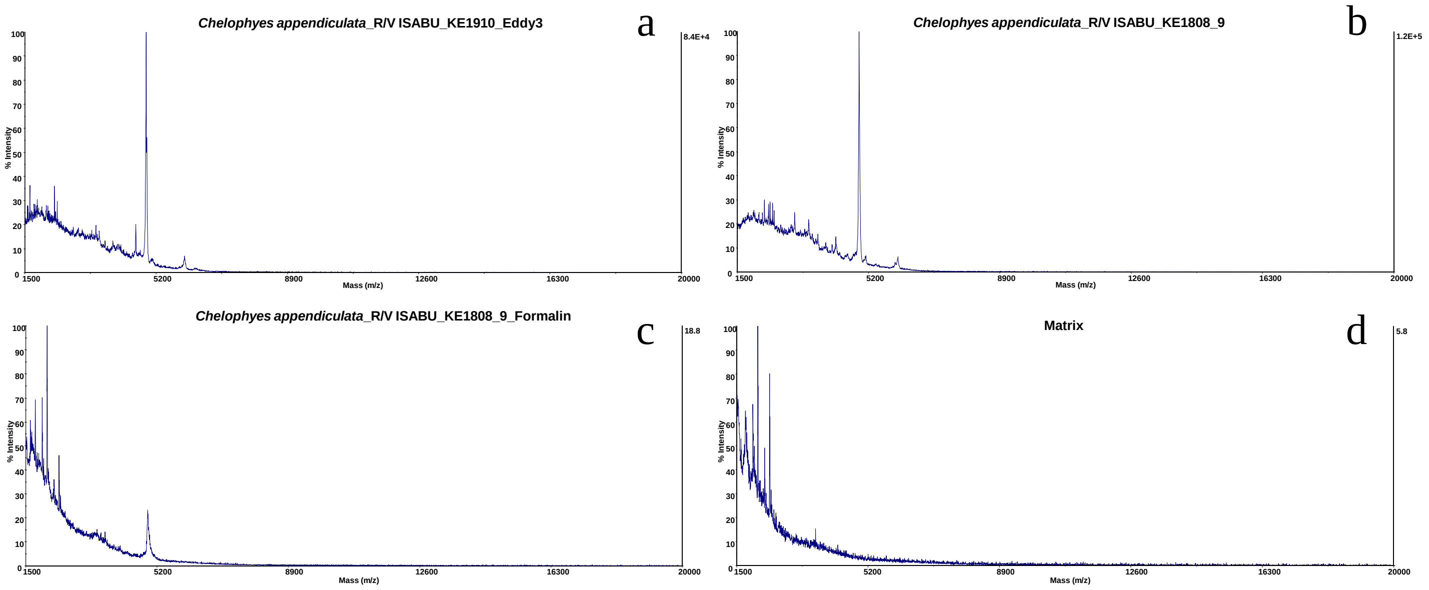

Figure S2. Raw protein mass spectra according to collection date, station, and fixation of the *C. appendiculata*. (a) Ethanol, Eddy3, 2019; (b) Ethanol, St.9, 2018; (c) Formalin, St.9, 2018; (d) Matrix-only.
