# Supplementary material for: Novel attempt at discrimination of a bullet-shaped siphonophore (Family Diphyidae) using matrix-assisted laser desorption/ionization time of flight mass spectrometry (MALDI-ToF MS)
Source: Sci Rep. 2021 Sep 24;11:19077. doi: 10.1038/s41598-021-98724-z (PMC8463557; doi:10.1038/s41598-021-98724-z)
Supplement: Supplementary file 4 — Supplementary Information 4. [file 41598_2021_98724_MOESM4_ESM.pdf]

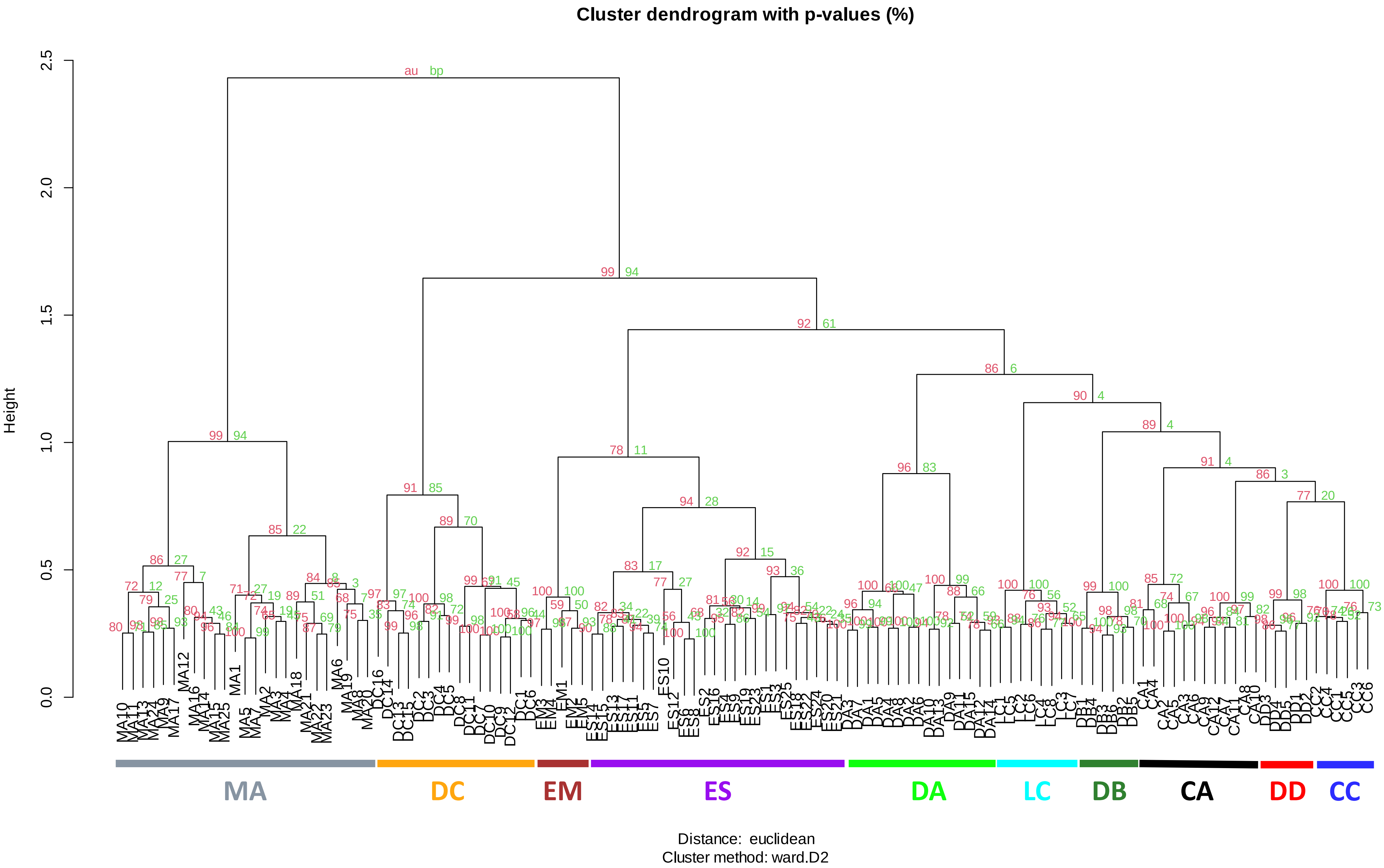


Figure S4. Cluster dendrogram with the p-values (%) of ten Diphyids (123 spectra). The number of each node dictates the AU (Red) and BP (Green).
