# Supplementary material for: Novel attempt at discrimination of a bullet-shaped siphonophore (Family Diphyidae) using matrix-assisted laser desorption/ionization time of flight mass spectrometry (MALDI-ToF MS)
Source: Sci Rep. 2021 Sep 24;11:19077. doi: 10.1038/s41598-021-98724-z (PMC8463557; doi:10.1038/s41598-021-98724-z)
Supplement: Supplementary file 5 — Supplementary Information 5. [file 41598_2021_98724_MOESM5_ESM.pdf]

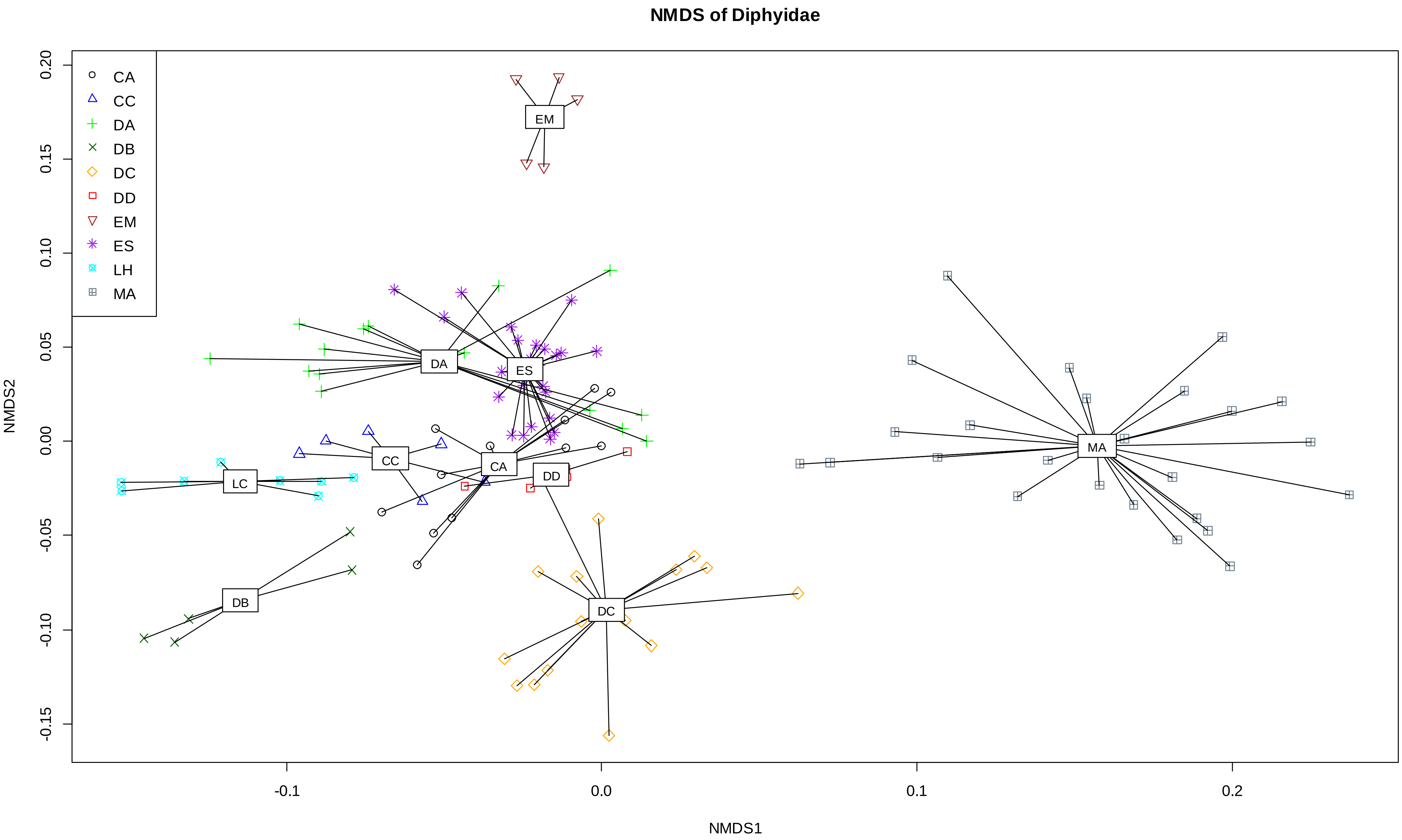

Figure S5. NMDS plot of the protein mass spectra of ten Diphyids from the Hellinger-transformed matrix (123 spectra).
