# Supplementary material for: Novel attempt at discrimination of a bullet-shaped siphonophore (Family Diphyidae) using matrix-assisted laser desorption/ionization time of flight mass spectrometry (MALDI-ToF MS)
Source: Sci Rep. 2021 Sep 24;11:19077. doi: 10.1038/s41598-021-98724-z (PMC8463557; doi:10.1038/s41598-021-98724-z)
Supplement: Supplementary file 6 — Supplementary Information 6. [file 41598_2021_98724_MOESM6_ESM.pdf]

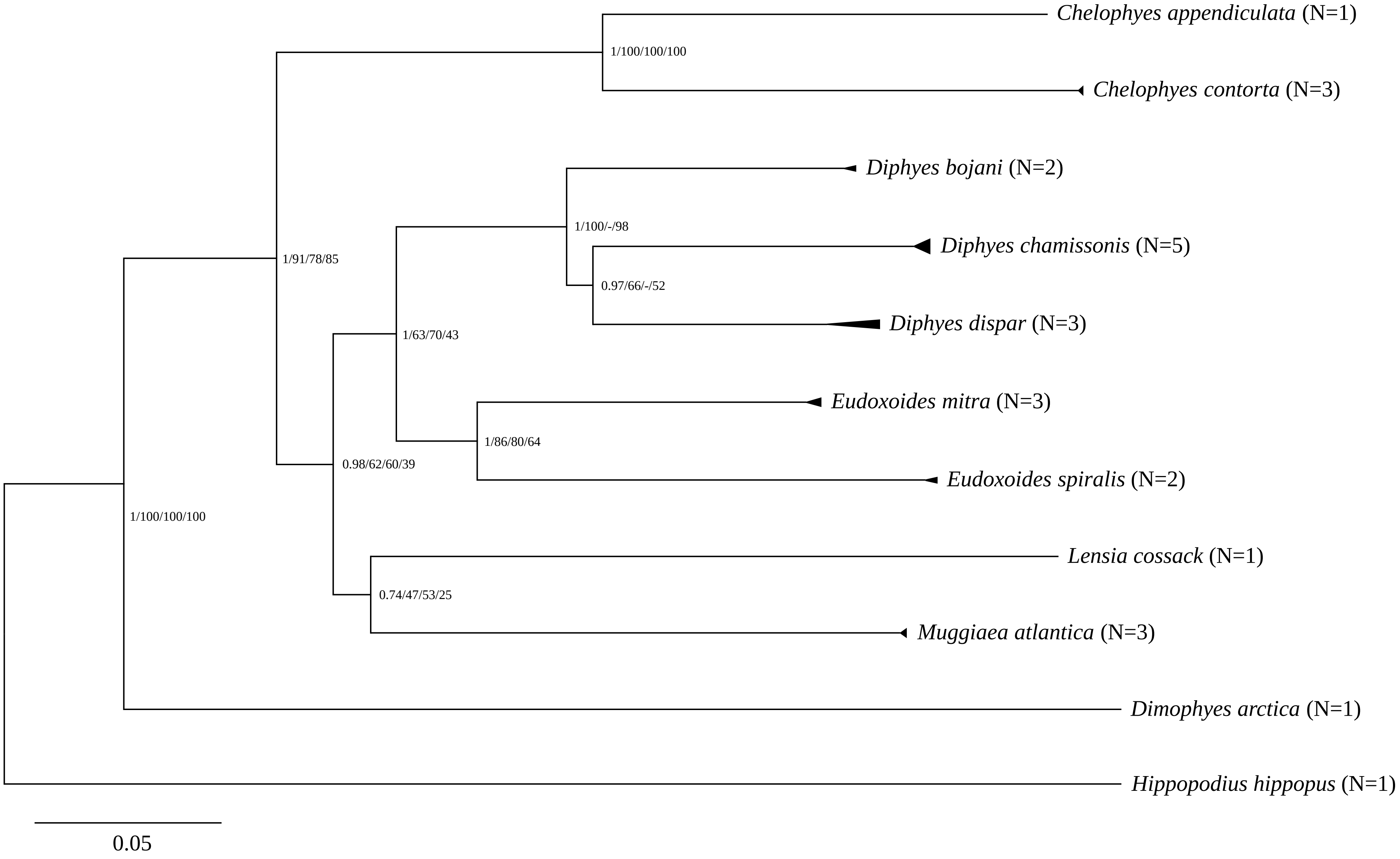

Figure S6. Molecular phylogenetic tree of Diphyidae species based on 25 mtCOI sequences. All positions containing gaps and missing data were eliminated. The number of each node dictates the BI/NJ/ML/MP bootstrap values. 'N' indicates the number of each sequence obtained in this study.
