# Supplementary material for: Novel attempt at discrimination of a bullet-shaped siphonophore (Family Diphyidae) using matrix-assisted laser desorption/ionization time of flight mass spectrometry (MALDI-ToF MS)
Source: Sci Rep. 2021 Sep 24;11:19077. doi: 10.1038/s41598-021-98724-z (PMC8463557; doi:10.1038/s41598-021-98724-z)
Supplement: Supplementary file 9 — Supplementary Information 9. [file 41598_2021_98724_MOESM9_ESM.pdf]

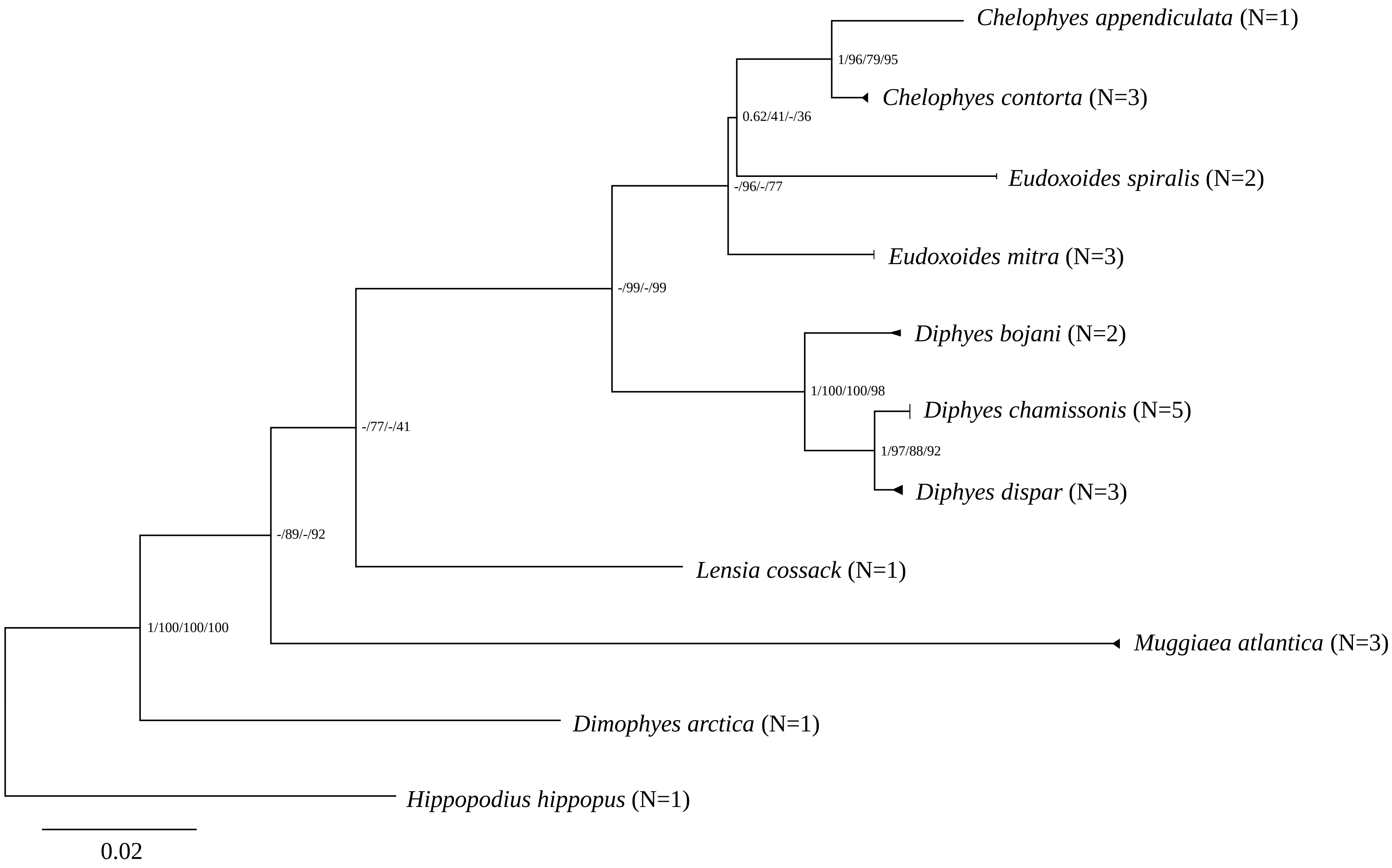

Figure S9. Molecular phylogenetic tree of Diphyidae species based on 25 ITS regions sequences. All positions containing gaps and missing data were eliminated. The number of each node dictates the BI/NJ/ML/MP bootstrap values. 'N' indicates the number of each sequence obtained in this study.
