# Supplementary material for: Novel attempt at discrimination of a bullet-shaped siphonophore (Family Diphyidae) using matrix-assisted laser desorption/ionization time of flight mass spectrometry (MALDI-ToF MS)
Source: Sci Rep. 2021 Sep 24;11:19077. doi: 10.1038/s41598-021-98724-z (PMC8463557; doi:10.1038/s41598-021-98724-z)
Supplement: Supplementary file 10 — Supplementary Information 10. [file 41598_2021_98724_MOESM10_ESM.pdf]

Table S1. K2P genetic distances of 25 concatenated sequences between Diphyidae species in this study. Standard error estimates are shown above the diagonal in italics.

Concatenated	1	2	3	4	5	6	7	8	9	10	11	12	13	14	15	16	17	18	19	20	21	22	23	24	25
1. *Hippopodius_hippopus*_KE1910_Eddy3_HH1		*0.008*	*0.008*	*0.008*	*0.008*	*0.008*	*0.008*	*0.008*	*0.008*	*0.008*	*0.008*	*0.008*	*0.008*	*0.008*	*0.008*	*0.008*	*0.008*	*0.008*	*0.008*	*0.008*	*0.008*	*0.008*	*0.007*	*0.007*	*0.007*
2. *Chelophyes_appendiculata*_KE1910_Eddy3_CA1	0.204		*0.004*	*0.004*	*0.004*	*0.008*	*0.007*	*0.007*	*0.006*	*0.006*	*0.006*	*0.006*	*0.006*	*0.007*	*0.006*	*0.007*	*0.006*	*0.006*	*0.006*	*0.006*	*0.006*	*0.007*	*0.007*	*0.007*	*0.007*
3. *Chelophyes_contorta*_KC2005_S09_CC1	0.200	0.063		*0.001*	*0.001*	*0.007*	*0.007*	*0.007*	*0.006*	*0.006*	*0.006*	*0.006*	*0.006*	*0.006*	*0.006*	*0.006*	*0.006*	*0.006*	*0.006*	*0.006*	*0.006*	*0.007*	*0.007*	*0.007*	*0.007*
4. *Chelophyes_contorta*_KC2005_S09_CC2	0.200	0.063	0.001		*0.000*	*0.007*	*0.007*	*0.007*	*0.006*	*0.006*	*0.006*	*0.006*	*0.006*	*0.006*	*0.006*	*0.006*	*0.006*	*0.006*	*0.006*	*0.006*	*0.006*	*0.007*	*0.007*	*0.007*	*0.007*
5. *Chelophyes_contorta*_KC2005_S09_CC3	0.200	0.063	0.001	0.001		*0.007*	*0.007*	*0.007*	*0.006*	*0.006*	*0.006*	*0.006*	*0.006*	*0.006*	*0.006*	*0.006*	*0.006*	*0.006*	*0.006*	*0.006*	*0.006*	*0.007*	*0.007*	*0.007*	*0.007*
6. *Dimophyes_arctica*_KE1910_St0_DA1	0.179	0.175	0.170	0.170	0.170		*0.008*	*0.008*	*0.008*	*0.008*	*0.008*	*0.008*	*0.008*	*0.008*	*0.008*	*0.008*	*0.007*	*0.007*	*0.007*	*0.007*	*0.007*	*0.008*	*0.008*	*0.008*	*0.008*
7. *Diphyes_bojani*_KE1808_St9_DB1	0.188	0.126	0.119	0.119	0.119	0.154		*0.001*	*0.004*	*0.004*	*0.004*	*0.004*	*0.004*	*0.004*	*0.004*	*0.004*	*0.005*	*0.005*	*0.005*	*0.006*	*0.006*	*0.007*	*0.006*	*0.006*	*0.006*
8. *Diphyes_bojani*_KE1808_St9_DB2	0.189	0.126	0.119	0.119	0.119	0.153	0.002		*0.004*	*0.004*	*0.004*	*0.004*	*0.004*	*0.004*	*0.004*	*0.004*	*0.005*	*0.005*	*0.006*	*0.006*	*0.006*	*0.007*	*0.006*	*0.006*	*0.006*
9. *Diphyes_chamissonis*_DB1809_St10_DC1	0.188	0.125	0.124	0.125	0.124	0.163	0.055	0.055		*0.000*	*0.001*	*0.001*	*0.001*	*0.004*	*0.004*	*0.004*	*0.005*	*0.005*	*0.005*	*0.006*	*0.006*	*0.007*	*0.006*	*0.006*	*0.006*
10. *Diphyes_chamissonis*_DB1809_St11_DC2	0.188	0.125	0.124	0.125	0.124	0.163	0.055	0.055	0.000		*0.001*	*0.001*	*0.001*	*0.004*	*0.004*	*0.004*	*0.005*	*0.005*	*0.005*	*0.006*	*0.006*	*0.007*	*0.006*	*0.006*	*0.006*
11. *Diphyes_chamissonis*_DB1809_St11_DC3	0.188	0.126	0.125	0.125	0.125	0.164	0.055	0.055	0.002	0.002		*0.001*	*0.001*	*0.004*	*0.004*	*0.004*	*0.005*	*0.005*	*0.005*	*0.006*	*0.006*	*0.007*	*0.006*	*0.006*	*0.006*
12. *Diphyes_chamissonis*_DB1809_St11_DC4	0.189	0.126	0.125	0.125	0.125	0.164	0.055	0.054	0.002	0.002	0.001		*0.001*	*0.004*	*0.004*	*0.004*	*0.005*	*0.005*	*0.005*	*0.006*	*0.006*	*0.007*	*0.006*	*0.006*	*0.006*
13. *Diphyes_chamissonis*_DB1809_St11_DC5	0.189	0.126	0.126	0.126	0.125	0.164	0.056	0.056	0.001	0.001	0.003	0.003		*0.004*	*0.004*	*0.004*	*0.005*	*0.005*	*0.005*	*0.006*	*0.006*	*0.007*	*0.006*	*0.006*	*0.006*
14. *Diphyes_dispar*_KE1710_St11.5_DD1	0.186	0.129	0.122	0.122	0.122	0.159	0.048	0.047	0.056	0.056	0.056	0.056	0.057		*0.001*	*0.002*	*0.005*	*0.005*	*0.005*	*0.006*	*0.006*	*0.007*	*0.007*	*0.007*	*0.007*
15. *Diphyes_dispar*_KE1710_St11.5_DD2	0.185	0.128	0.121	0.122	0.121	0.159	0.049	0.048	0.054	0.054	0.055	0.055	0.056	0.004		*0.001*	*0.006*	*0.006*	*0.006*	*0.005*	*0.006*	*0.007*	*0.007*	*0.007*	*0.007*
16. *Diphyes_dispar*_KE1710_St11.5_DD3	0.182	0.129	0.122	0.123	0.122	0.158	0.047	0.046	0.053	0.053	0.054	0.053	0.054	0.008	0.004		*0.005*	*0.005*	*0.006*	*0.005*	*0.006*	*0.007*	*0.007*	*0.007*	*0.007*
17. *Eudoxoides_mitra*_KC2005_S12_EM1	0.175	0.111	0.114	0.114	0.114	0.162	0.082	0.083	0.093	0.093	0.094	0.093	0.093	0.087	0.088	0.087		*0.000*	*0.001*	*0.005*	*0.005*	*0.006*	*0.006*	*0.006*	*0.006*
18. *Eudoxoides_mitra*_KC2005_S12_EM2	0.175	0.111	0.114	0.114	0.114	0.162	0.082	0.083	0.093	0.093	0.094	0.093	0.093	0.087	0.088	0.087	0.000		*0.001*	*0.005*	*0.005*	*0.006*	*0.006*	*0.006*	*0.006*
19. *Eudoxoides_mitra*_KC2005_S12_EM3	0.175	0.111	0.115	0.115	0.115	0.161	0.082	0.083	0.092	0.092	0.093	0.093	0.093	0.088	0.089	0.087	0.002	0.002		*0.005*	*0.005*	*0.007*	*0.006*	*0.006*	*0.006*
20. *Eudoxoides_spiralis*_KE1808_St8_ES1	0.190	0.118	0.116	0.115	0.116	0.162	0.091	0.091	0.104	0.104	0.104	0.105	0.104	0.098	0.098	0.098	0.079	0.079	0.079		*0.001*	*0.006*	*0.007*	*0.007*	*0.007*
21. *Eudoxoides_spiralis*_KE1910_Eddy3_ES2	0.190	0.117	0.116	0.115	0.116	0.161	0.090	0.091	0.104	0.104	0.104	0.104	0.104	0.097	0.097	0.097	0.079	0.079	0.079	0.002		*0.006*	*0.007*	*0.007*	*0.007*
22. *Lensia_cossack*_KE1910_Eddy1_LC1	0.190	0.136	0.133	0.133	0.132	0.155	0.125	0.125	0.125	0.125	0.125	0.125	0.124	0.125	0.125	0.125	0.117	0.117	0.117	0.126	0.126		*0.006*	*0.006*	*0.006*
23. *Muggiaea_atlantica*_DB1804_St14_MA1	0.183	0.149	0.139	0.139	0.139	0.159	0.116	0.115	0.119	0.119	0.120	0.120	0.121	0.117	0.115	0.115	0.114	0.114	0.113	0.118	0.118	0.124		*0.001*	*0.001*
24. *Muggiaea_atlantica*_DB1804_St14_MA2	0.183	0.148	0.139	0.139	0.139	0.159	0.115	0.114	0.119	0.119	0.120	0.120	0.121	0.117	0.115	0.115	0.114	0.114	0.113	0.117	0.117	0.123	0.001		*0.000*
25. *Muggiaea_atlantica*_DB1804_St14_MA3	0.183	0.148	0.139	0.139	0.139	0.159	0.115	0.114	0.119	0.119	0.120	0.120	0.121	0.117	0.115	0.115	0.114	0.114	0.113	0.117	0.117	0.123	0.001	0.000	
