# Supplementary material for: Novel attempt at discrimination of a bullet-shaped siphonophore (Family Diphyidae) using matrix-assisted laser desorption/ionization time of flight mass spectrometry (MALDI-ToF MS)
Source: Sci Rep. 2021 Sep 24;11:19077. doi: 10.1038/s41598-021-98724-z (PMC8463557; doi:10.1038/s41598-021-98724-z)
Supplement: Supplementary file 11 — Supplementary Information 11. [file 41598_2021_98724_MOESM11_ESM.pdf]

Table S2. K2P genetic distances of 25 mtCOI sequences between Diphyidae species in this study. Standard error estimates are shown above the diagonal in italics.

mtCOI	1	2	3	4	5	6	7	8	9	10	11	12	13	14	15	16	17	18	19	20	21	22	23	24	25
1. *Hippopodius_hippopus*_KE1910_Eddy3_HH1		*0.040*	*0.040*	*0.040*	*0.040*	*0.039*	*0.037*	*0.037*	*0.036*	*0.036*	*0.036*	*0.037*	*0.037*	*0.036*	*0.036*	*0.035*	*0.032*	*0.032*	*0.033*	*0.039*	*0.039*	*0.043*	*0.039*	*0.040*	*0.040*
2. *Chelophyes_appendiculata*_KE1910_Eddy3_CA1	0.601		*0.023*	*0.023*	*0.023*	*0.038*	*0.029*	*0.029*	*0.026*	*0.026*	*0.026*	*0.026*	*0.026*	*0.029*	*0.029*	*0.028*	*0.027*	*0.027*	*0.027*	*0.027*	*0.027*	*0.030*	*0.032*	*0.032*	*0.032*
3. *Chelophyes_contorta*_KC2005_S09_CC1	0.590	0.249		*0.002*	*0.002*	*0.036*	*0.029*	*0.029*	*0.028*	*0.028*	*0.029*	*0.029*	*0.029*	*0.028*	*0.028*	*0.029*	*0.028*	*0.028*	*0.029*	*0.029*	*0.029*	*0.032*	*0.029*	*0.029*	*0.029*
4. *Chelophyes_contorta*_KC2005_S09_CC2	0.590	0.247	0.002		*0.002*	*0.036*	*0.029*	*0.029*	*0.028*	*0.028*	*0.029*	*0.029*	*0.029*	*0.028*	*0.028*	*0.029*	*0.028*	*0.028*	*0.029*	*0.029*	*0.029*	*0.032*	*0.029*	*0.029*	*0.029*
5. *Chelophyes_contorta*_KC2005_S09_CC3	0.590	0.251	0.002	0.003		*0.037*	*0.029*	*0.029*	*0.028*	*0.028*	*0.029*	*0.029*	*0.029*	*0.028*	*0.028*	*0.029*	*0.028*	*0.028*	*0.029*	*0.029*	*0.029*	*0.032*	*0.029*	*0.029*	*0.029*
6. *Dimophyes_arctica*_KE1910_St0_DA1	0.602	0.523	0.503	0.506	0.506		*0.034*	*0.034*	*0.036*	*0.036*	*0.036*	*0.036*	*0.037*	*0.036*	*0.036*	*0.035*	*0.035*	*0.035*	*0.035*	*0.038*	*0.037*	*0.037*	*0.035*	*0.035*	*0.035*
7. *Diphyes_bojani*_KE1808_St9_DB1	0.552	0.376	0.370	0.372	0.370	0.453		*0.003*	*0.018*	*0.018*	*0.017*	*0.018*	*0.018*	*0.018*	*0.019*	*0.018*	*0.020*	*0.020*	*0.020*	*0.022*	*0.021*	*0.026*	*0.025*	*0.024*	*0.024*
8. *Diphyes_bojani*_KE1808_St9_DB2	0.555	0.373	0.367	0.370	0.367	0.447	0.006		*0.018*	*0.018*	*0.017*	*0.018*	*0.018*	*0.018*	*0.018*	*0.018*	*0.020*	*0.020*	*0.020*	*0.022*	*0.022*	*0.026*	*0.024*	*0.024*	*0.024*
9. *Diphyes_chamissonis*_DB1809_St10_DC1	0.558	0.354	0.380	0.383	0.378	0.493	0.173	0.173		*0.000*	*0.003*	*0.003*	*0.003*	*0.020*	*0.019*	*0.018*	*0.022*	*0.022*	*0.021*	*0.026*	*0.025*	*0.027*	*0.027*	*0.027*	*0.027*
10. *Diphyes_chamissonis*_DB1809_St11_DC2	0.558	0.354	0.380	0.383	0.378	0.493	0.173	0.173	0.000		*0.003*	*0.003*	*0.003*	*0.020*	*0.019*	*0.018*	*0.022*	*0.022*	*0.021*	*0.026*	*0.025*	*0.027*	*0.027*	*0.027*	*0.027*
11. *Diphyes_chamissonis*_DB1809_St11_DC3	0.552	0.355	0.383	0.386	0.381	0.499	0.169	0.169	0.008	0.008		*0.002*	*0.004*	*0.019*	*0.018*	*0.018*	*0.021*	*0.021*	*0.021*	*0.026*	*0.025*	*0.027*	*0.027*	*0.027*	*0.027*
12. *Diphyes_chamissonis*_DB1809_St11_DC4	0.558	0.357	0.383	0.386	0.380	0.497	0.169	0.169	0.008	0.008	0.003		*0.004*	*0.019*	*0.019*	*0.018*	*0.022*	*0.022*	*0.022*	*0.026*	*0.025*	*0.027*	*0.027*	*0.027*	*0.027*
13. *Diphyes_chamissonis*_DB1809_St11_DC5	0.565	0.357	0.392	0.394	0.389	0.503	0.177	0.177	0.006	0.006	0.011	0.011		*0.020*	*0.019*	*0.018*	*0.022*	*0.022*	*0.022*	*0.026*	*0.026*	*0.027*	*0.027*	*0.028*	*0.028*
14. *Diphyes_dispar*_KE1710_St11.5_DD1	0.549	0.383	0.368	0.371	0.368	0.483	0.174	0.170	0.176	0.176	0.176	0.178	0.185		*0.005*	*0.007*	*0.021*	*0.021*	*0.021*	*0.025*	*0.024*	*0.028*	*0.026*	*0.027*	*0.027*
15. *Diphyes_dispar*_KE1710_St11.5_DD2	0.536	0.377	0.365	0.368	0.365	0.476	0.176	0.172	0.168	0.168	0.168	0.170	0.176	0.017		*0.005*	*0.021*	*0.021*	*0.022*	*0.025*	*0.024*	*0.028*	*0.025*	*0.026*	*0.026*
16. *Diphyes_dispar*_KE1710_St11.5_DD3	0.517	0.379	0.373	0.376	0.373	0.467	0.163	0.159	0.159	0.159	0.161	0.161	0.168	0.037	0.019		*0.021*	*0.021*	*0.021*	*0.025*	*0.024*	*0.028*	*0.025*	*0.026*	*0.026*
17. *Eudoxoides_mitra*_KC2005_S12_EM1	0.454	0.350	0.388	0.388	0.391	0.491	0.231	0.235	0.256	0.256	0.261	0.261	0.261	0.238	0.242	0.238		*0.000*	*0.003*	*0.020*	*0.020*	*0.026*	*0.024*	*0.024*	*0.024*
18. *Eudoxoides_mitra*_KC2005_S12_EM2	0.454	0.350	0.388	0.388	0.391	0.491	0.231	0.235	0.256	0.256	0.261	0.261	0.261	0.238	0.242	0.238	0.000		*0.003*	*0.020*	*0.020*	*0.026*	*0.024*	*0.024*	*0.024*
19. *Eudoxoides_mitra*_KC2005_S12_EM3	0.457	0.350	0.394	0.394	0.396	0.485	0.229	0.233	0.251	0.251	0.256	0.256	0.256	0.242	0.247	0.238	0.008	0.008		*0.020*	*0.020*	*0.026*	*0.024*	*0.024*	*0.024*
20. *Eudoxoides_spiralis*_KE1808_St8_ES1	0.565	0.372	0.393	0.390	0.396	0.512	0.264	0.267	0.314	0.314	0.312	0.317	0.314	0.295	0.295	0.292	0.218	0.218	0.218		*0.003*	*0.028*	*0.026*	*0.026*	*0.026*
21. *Eudoxoides_spiralis*_KE1910_Eddy3_ES2	0.565	0.367	0.390	0.387	0.393	0.505	0.257	0.260	0.307	0.307	0.304	0.309	0.312	0.287	0.287	0.285	0.216	0.216	0.216	0.006		*0.028*	*0.026*	*0.026*	*0.026*
22. *Lensia_cossack*_KE1910_Eddy1_LC1	0.593	0.414	0.402	0.405	0.402	0.514	0.340	0.343	0.338	0.338	0.336	0.340	0.335	0.348	0.345	0.343	0.353	0.353	0.348	0.370	0.368		*0.027*	*0.027*	*0.027*
23. *Muggiaea_atlantica*_DB1804_St14_MA1	0.556	0.417	0.381	0.383	0.381	0.465	0.303	0.298	0.325	0.325	0.328	0.327	0.335	0.300	0.288	0.285	0.274	0.274	0.270	0.299	0.297	0.331		*0.002*	*0.002*
24. *Muggiaea_atlantica*_DB1804_St14_MA2	0.556	0.414	0.383	0.386	0.383	0.465	0.298	0.294	0.327	0.327	0.330	0.330	0.338	0.302	0.290	0.287	0.277	0.277	0.272	0.294	0.292	0.329	0.003		*0.000*
25. *Muggiaea_atlantica*_DB1804_St14_MA3	0.556	0.414	0.383	0.386	0.383	0.465	0.298	0.294	0.327	0.327	0.330	0.330	0.338	0.302	0.290	0.287	0.277	0.277	0.272	0.294	0.292	0.329	0.003	0.000	
