# Supplementary material for: Novel attempt at discrimination of a bullet-shaped siphonophore (Family Diphyidae) using matrix-assisted laser desorption/ionization time of flight mass spectrometry (MALDI-ToF MS)
Source: Sci Rep. 2021 Sep 24;11:19077. doi: 10.1038/s41598-021-98724-z (PMC8463557; doi:10.1038/s41598-021-98724-z)
Supplement: Supplementary file 12 — Supplementary Information 12. [file 41598_2021_98724_MOESM12_ESM.pdf]

Table S3. K2P genetic distances of 25 16S rRNA sequences between Diphyidae species in this study. Standard error estimates are shown above the diagonal in italics.

16S rRNA	1	2	3	4	5	6	7	8	9	10	11	12	13	14	15	16	17	18	19	20	21	22	23	24	25
1. *Hippopodius_hippopus*_KE1910_Eddy3_HH1		*0.092*	*0.079*	*0.079*	*0.079*	*0.049*	*0.060*	*0.060*	*0.057*	*0.057*	*0.058*	*0.058*	*0.057*	*0.054*	*0.054*	*0.054*	*0.062*	*0.062*	*0.062*	*0.061*	*0.062*	*0.054*	*0.048*	*0.048*	*0.048*
2. *Chelophyes_appendiculata*_KE1910_Eddy3_CA1	0.878		*0.016*	*0.016*	*0.016*	*0.049*	*0.035*	*0.035*	*0.038*	*0.038*	*0.038*	*0.038*	*0.038*	*0.037*	*0.037*	*0.037*	*0.031*	*0.031*	*0.031*	*0.034*	*0.034*	*0.038*	*0.040*	*0.040*	*0.040*
3. *Chelophyes_contorta*_KC2005_S09_CC1	0.830	0.106		*0.003*	*0.003*	*0.046*	*0.032*	*0.032*	*0.036*	*0.036*	*0.037*	*0.037*	*0.036*	*0.035*	*0.035*	*0.035*	*0.030*	*0.030*	*0.030*	*0.031*	*0.032*	*0.035*	*0.035*	*0.035*	*0.035*
4. *Chelophyes_contorta*_KC2005_S09_CC2	0.830	0.109	0.004		*0.000*	*0.046*	*0.032*	*0.032*	*0.037*	*0.037*	*0.037*	*0.037*	*0.037*	*0.035*	*0.035*	*0.035*	*0.030*	*0.030*	*0.030*	*0.031*	*0.032*	*0.035*	*0.034*	*0.034*	*0.034*
5. *Chelophyes_contorta*_KC2005_S09_CC3	0.830	0.109	0.004	0.000		*0.046*	*0.032*	*0.032*	*0.037*	*0.037*	*0.037*	*0.037*	*0.037*	*0.035*	*0.035*	*0.035*	*0.030*	*0.030*	*0.030*	*0.031*	*0.032*	*0.035*	*0.034*	*0.034*	*0.034*
6. *Dimophyes_arctica*_KE1910_St0_DA1	0.567	0.585	0.558	0.558	0.558		*0.039*	*0.039*	*0.041*	*0.041*	*0.041*	*0.041*	*0.041*	*0.041*	*0.041*	*0.041*	*0.041*	*0.041*	*0.041*	*0.038*	*0.038*	*0.034*	*0.037*	*0.037*	*0.037*
7. *Diphyes_bojani*_KE1808_St9_DB1	0.688	0.396	0.352	0.356	0.356	0.461		*0.000*	*0.017*	*0.017*	*0.017*	*0.017*	*0.017*	*0.015*	*0.015*	*0.015*	*0.023*	*0.023*	*0.023*	*0.025*	*0.026*	*0.034*	*0.027*	*0.027*	*0.027*
8. *Diphyes_bojani*_KE1808_St9_DB2	0.688	0.396	0.352	0.356	0.356	0.461	0.000		*0.017*	*0.017*	*0.017*	*0.017*	*0.017*	*0.015*	*0.015*	*0.015*	*0.023*	*0.023*	*0.023*	*0.025*	*0.026*	*0.034*	*0.027*	*0.027*	*0.027*
9. *Diphyes_chamissonis*_DB1809_St10_DC1	0.651	0.423	0.388	0.392	0.392	0.490	0.133	0.133		*0.000*	*0.002*	*0.003*	*0.000*	*0.019*	*0.019*	*0.019*	*0.026*	*0.026*	*0.026*	*0.028*	*0.028*	*0.034*	*0.027*	*0.027*	*0.027*
10. *Diphyes_chamissonis*_DB1809_St11_DC2	0.651	0.423	0.388	0.392	0.392	0.490	0.133	0.133	0.000		*0.002*	*0.003*	*0.000*	*0.019*	*0.019*	*0.019*	*0.026*	*0.026*	*0.026*	*0.028*	*0.028*	*0.034*	*0.027*	*0.027*	*0.027*
11. *Diphyes_chamissonis*_DB1809_St11_DC3	0.656	0.427	0.392	0.395	0.395	0.494	0.135	0.135	0.002	0.002		*0.002*	*0.002*	*0.020*	*0.019*	*0.019*	*0.027*	*0.027*	*0.027*	*0.028*	*0.028*	*0.035*	*0.027*	*0.027*	*0.027*
12. *Diphyes_chamissonis*_DB1809_St11_DC4	0.656	0.427	0.392	0.395	0.395	0.490	0.133	0.133	0.004	0.004	0.002		*0.003*	*0.019*	*0.019*	*0.019*	*0.026*	*0.026*	*0.026*	*0.028*	*0.028*	*0.035*	*0.027*	*0.027*	*0.027*
13. *Diphyes_chamissonis*_DB1809_St11_DC5	0.651	0.423	0.388	0.392	0.392	0.490	0.133	0.133	0.000	0.000	0.002	0.004		*0.019*	*0.019*	*0.019*	*0.026*	*0.026*	*0.026*	*0.028*	*0.028*	*0.034*	*0.027*	*0.027*	*0.027*
14. *Diphyes_dispar*_KE1710_St11.5_DD1	0.651	0.419	0.381	0.385	0.385	0.474	0.089	0.089	0.157	0.157	0.160	0.157	0.157		*0.002*	*0.002*	*0.024*	*0.024*	*0.024*	*0.026*	*0.026*	*0.034*	*0.028*	*0.028*	*0.028*
15. *Diphyes_dispar*_KE1710_St11.5_DD2	0.651	0.419	0.377	0.381	0.381	0.474	0.089	0.089	0.154	0.154	0.157	0.154	0.154	0.002		*0.000*	*0.024*	*0.024*	*0.024*	*0.026*	*0.026*	*0.034*	*0.028*	*0.028*	*0.028*
16. *Diphyes_dispar*_KE1710_St11.5_DD3	0.651	0.419	0.377	0.381	0.381	0.474	0.089	0.089	0.154	0.154	0.157	0.154	0.154	0.002	0.000		*0.024*	*0.024*	*0.024*	*0.026*	*0.026*	*0.034*	*0.028*	*0.028*	*0.028*
17. *Eudoxoides_mitra*_KC2005_S12_EM1	0.712	0.343	0.335	0.335	0.335	0.488	0.221	0.221	0.270	0.270	0.273	0.270	0.270	0.248	0.248	0.248		*0.000*	*0.000*	*0.024*	*0.024*	*0.029*	*0.027*	*0.027*	*0.027*
18. *Eudoxoides_mitra*_KC2005_S12_EM2	0.712	0.343	0.335	0.335	0.335	0.488	0.221	0.221	0.270	0.270	0.273	0.270	0.270	0.248	0.248	0.248	0.000		*0.000*	*0.024*	*0.024*	*0.029*	*0.027*	*0.027*	*0.027*
19. *Eudoxoides_mitra*_KC2005_S12_EM3	0.712	0.343	0.335	0.335	0.335	0.488	0.221	0.221	0.270	0.270	0.273	0.270	0.270	0.248	0.248	0.248	0.000	0.000		*0.024*	*0.024*	*0.029*	*0.027*	*0.027*	*0.027*
20. *Eudoxoides_spiralis*_KE1808_St8_ES1	0.695	0.357	0.322	0.322	0.322	0.433	0.238	0.238	0.272	0.272	0.276	0.272	0.272	0.247	0.243	0.243	0.230	0.230	0.230		*0.002*	*0.031*	*0.027*	*0.027*	*0.027*
21. *Eudoxoides_spiralis*_KE1910_Eddy3_ES2	0.702	0.354	0.326	0.326	0.326	0.433	0.241	0.241	0.276	0.276	0.279	0.276	0.276	0.250	0.247	0.247	0.233	0.233	0.233	0.002		*0.032*	*0.027*	*0.027*	*0.027*
22. *Lensia_cossack*_KE1910_Eddy1_LC1	0.639	0.402	0.375	0.375	0.375	0.399	0.363	0.363	0.363	0.363	0.366	0.366	0.363	0.366	0.366	0.366	0.293	0.293	0.293	0.330	0.333		*0.030*	*0.030*	*0.030*
23. *Muggiaea_atlantica*_DB1804_St14_MA1	0.568	0.409	0.366	0.362	0.362	0.432	0.273	0.273	0.260	0.260	0.263	0.263	0.260	0.289	0.286	0.286	0.266	0.266	0.266	0.257	0.260	0.318		*0.000*	*0.000*
24. *Muggiaea_atlantica*_DB1804_St14_MA2	0.568	0.409	0.366	0.362	0.362	0.432	0.273	0.273	0.260	0.260	0.263	0.263	0.260	0.289	0.286	0.286	0.266	0.266	0.266	0.257	0.260	0.318	0.000		*0.000*
25. *Muggiaea_atlantica*_DB1804_St14_MA3	0.568	0.409	0.366	0.362	0.362	0.432	0.273	0.273	0.260	0.260	0.263	0.263	0.260	0.289	0.286	0.286	0.266	0.266	0.266	0.257	0.260	0.318	0.000	0.000	
