# Supplementary material for: Novel attempt at discrimination of a bullet-shaped siphonophore (Family Diphyidae) using matrix-assisted laser desorption/ionization time of flight mass spectrometry (MALDI-ToF MS)
Source: Sci Rep. 2021 Sep 24;11:19077. doi: 10.1038/s41598-021-98724-z (PMC8463557; doi:10.1038/s41598-021-98724-z)
Supplement: Supplementary file 13 — Supplementary Information 13. [file 41598_2021_98724_MOESM13_ESM.pdf]

Table S4. K2P genetic distances of 25 18S rRNA sequences between Diphyidae species in this study. Standard error estimates are shown above the diagonal in italics.

18S rRNA	1	2	3	4	5	6	7	8	9	10	11	12	13	14	15	16	17	18	19	20	21	22	23	24	25
1. *Hippopodius_hippopus*_KE1910_Eddy3_HH1		*0.001*	*0.001*	*0.001*	*0.001*	*0.001*	*0.001*	*0.001*	*0.001*	*0.001*	*0.001*	*0.001*	*0.001*	*0.001*	*0.001*	*0.001*	*0.001*	*0.001*	*0.001*	*0.001*	*0.001*	*0.002*	*0.001*	*0.001*	*0.001*
2. *Chelophyes_appendiculata*_KE1910_Eddy3_CA1	0.002		*0.000*	*0.000*	*0.000*	*0.001*	*0.000*	*0.000*	*0.001*	*0.001*	*0.001*	*0.001*	*0.001*	*0.000*	*0.000*	*0.000*	*0.001*	*0.001*	*0.001*	*0.001*	*0.001*	*0.002*	*0.001*	*0.001*	*0.001*
3. *Chelophyes_contorta*_KC2005_S09_CC1	0.002	0.000		*0.000*	*0.000*	*0.001*	*0.000*	*0.000*	*0.001*	*0.001*	*0.001*	*0.001*	*0.001*	*0.000*	*0.000*	*0.000*	*0.001*	*0.001*	*0.001*	*0.001*	*0.001*	*0.002*	*0.001*	*0.001*	*0.001*
4. *Chelophyes_contorta*_KC2005_S09_CC2	0.002	0.000	0.000		*0.000*	*0.001*	*0.000*	*0.000*	*0.001*	*0.001*	*0.001*	*0.001*	*0.001*	*0.000*	*0.000*	*0.000*	*0.001*	*0.001*	*0.001*	*0.001*	*0.001*	*0.002*	*0.001*	*0.001*	*0.001*
5. *Chelophyes_contorta*_KC2005_S09_CC3	0.002	0.000	0.000	0.000		*0.001*	*0.000*	*0.000*	*0.001*	*0.001*	*0.001*	*0.001*	*0.001*	*0.000*	*0.000*	*0.000*	*0.001*	*0.001*	*0.001*	*0.001*	*0.001*	*0.002*	*0.001*	*0.001*	*0.001*
6. *Dimophyes_arctica*_KE1910_St0_DA1	0.002	0.002	0.002	0.002	0.002		*0.001*	*0.001*	*0.001*	*0.001*	*0.001*	*0.001*	*0.001*	*0.001*	*0.001*	*0.001*	*0.001*	*0.001*	*0.001*	*0.001*	*0.001*	*0.002*	*0.002*	*0.002*	*0.002*
7. *Diphyes_bojani*_KE1808_St9_DB1	0.002	0.000	0.000	0.000	0.000	0.002		*0.000*	*0.001*	*0.001*	*0.001*	*0.001*	*0.001*	*0.000*	*0.000*	*0.000*	*0.001*	*0.001*	*0.001*	*0.001*	*0.001*	*0.002*	*0.001*	*0.001*	*0.001*
8. *Diphyes_bojani*_KE1808_St9_DB2	0.002	0.000	0.000	0.000	0.000	0.002	0.000		*0.001*	*0.001*	*0.001*	*0.001*	*0.001*	*0.000*	*0.000*	*0.000*	*0.001*	*0.001*	*0.001*	*0.001*	*0.001*	*0.002*	*0.001*	*0.001*	*0.001*
9. *Diphyes_chamissonis*_DB1809_St10_DC1	0.004	0.001	0.001	0.001	0.001	0.004	0.001	0.001		*0.000*	*0.000*	*0.000*	*0.000*	*0.001*	*0.001*	*0.001*	*0.001*	*0.001*	*0.001*	*0.001*	*0.001*	*0.002*	*0.001*	*0.001*	*0.001*
10. *Diphyes_chamissonis*_DB1809_St11_DC2	0.004	0.001	0.001	0.001	0.001	0.004	0.001	0.001	0.000		*0.000*	*0.000*	*0.000*	*0.001*	*0.001*	*0.001*	*0.001*	*0.001*	*0.001*	*0.001*	*0.001*	*0.002*	*0.001*	*0.001*	*0.001*
11. *Diphyes_chamissonis*_DB1809_St11_DC3	0.004	0.001	0.001	0.001	0.001	0.004	0.001	0.001	0.000	0.000		*0.000*	*0.000*	*0.001*	*0.001*	*0.001*	*0.001*	*0.001*	*0.001*	*0.001*	*0.001*	*0.002*	*0.001*	*0.001*	*0.001*
12. *Diphyes_chamissonis*_DB1809_St11_DC4	0.004	0.001	0.001	0.001	0.001	0.004	0.001	0.001	0.000	0.000	0.000		*0.000*	*0.001*	*0.001*	*0.001*	*0.001*	*0.001*	*0.001*	*0.001*	*0.001*	*0.002*	*0.001*	*0.001*	*0.001*
13. *Diphyes_chamissonis*_DB1809_St11_DC5	0.004	0.001	0.001	0.001	0.001	0.004	0.001	0.001	0.000	0.000	0.000	0.000		*0.001*	*0.001*	*0.001*	*0.001*	*0.001*	*0.001*	*0.001*	*0.001*	*0.002*	*0.001*	*0.001*	*0.001*
14. *Diphyes_dispar*_KE1710_St11.5_DD1	0.002	0.000	0.000	0.000	0.000	0.002	0.000	0.000	0.001	0.001	0.001	0.001	0.001		*0.000*	*0.000*	*0.001*	*0.001*	*0.001*	*0.001*	*0.001*	*0.002*	*0.001*	*0.001*	*0.001*
15. *Diphyes_dispar*_KE1710_St11.5_DD2	0.002	0.000	0.000	0.000	0.000	0.002	0.000	0.000	0.001	0.001	0.001	0.001	0.001	0.000		*0.000*	*0.001*	*0.001*	*0.001*	*0.001*	*0.001*	*0.002*	*0.001*	*0.001*	*0.001*
16. *Diphyes_dispar*_KE1710_St11.5_DD3	0.002	0.000	0.000	0.000	0.000	0.002	0.000	0.000	0.001	0.001	0.001	0.001	0.001	0.000	0.000		*0.001*	*0.001*	*0.001*	*0.001*	*0.001*	*0.002*	*0.001*	*0.001*	*0.001*
17. *Eudoxoides_mitra*_KC2005_S12_EM1	0.003	0.001	0.001	0.001	0.001	0.003	0.001	0.001	0.002	0.002	0.002	0.002	0.002	0.001	0.001	0.001		*0.000*	*0.000*	*0.001*	*0.001*	*0.002*	*0.001*	*0.001*	*0.001*
18. *Eudoxoides_mitra*_KC2005_S12_EM2	0.003	0.001	0.001	0.001	0.001	0.003	0.001	0.001	0.002	0.002	0.002	0.002	0.002	0.001	0.001	0.001	0.000		*0.000*	*0.001*	*0.001*	*0.002*	*0.001*	*0.001*	*0.001*
19. *Eudoxoides_mitra*_KC2005_S12_EM3	0.003	0.001	0.001	0.001	0.001	0.003	0.001	0.001	0.002	0.002	0.002	0.002	0.002	0.001	0.001	0.001	0.000	0.000		*0.001*	*0.001*	*0.002*	*0.001*	*0.001*	*0.001*
20. *Eudoxoides_spiralis*_KE1808_St8_ES1	0.003	0.001	0.001	0.001	0.001	0.003	0.001	0.001	0.002	0.002	0.002	0.002	0.002	0.001	0.001	0.001	0.001	0.001	0.001		*0.000*	*0.002*	*0.001*	*0.001*	*0.001*
21. *Eudoxoides_spiralis*_KE1910_Eddy3_ES2	0.003	0.001	0.001	0.001	0.001	0.003	0.001	0.001	0.002	0.002	0.002	0.002	0.002	0.001	0.001	0.001	0.001	0.001	0.001	0.000		*0.002*	*0.001*	*0.001*	*0.001*
22. *Lensia_cossack*_KE1910_Eddy1_LC1	0.006	0.004	0.004	0.004	0.004	0.006	0.004	0.004	0.005	0.005	0.005	0.005	0.005	0.004	0.004	0.004	0.004	0.004	0.004	0.004	0.004		*0.002*	*0.002*	*0.002*
23. *Muggiaea_atlantica*_DB1804_St14_MA1	0.004	0.002	0.002	0.002	0.002	0.005	0.002	0.002	0.004	0.004	0.004	0.004	0.004	0.002	0.002	0.002	0.003	0.003	0.003	0.003	0.003	0.005		*0.000*	*0.000*
24. *Muggiaea_atlantica*_DB1804_St14_MA2	0.004	0.002	0.002	0.002	0.002	0.005	0.002	0.002	0.004	0.004	0.004	0.004	0.004	0.002	0.002	0.002	0.003	0.003	0.003	0.003	0.003	0.005	0.000		*0.000*
25. *Muggiaea_atlantica*_DB1804_St14_MA3	0.004	0.002	0.002	0.002	0.002	0.005	0.002	0.002	0.004	0.004	0.004	0.004	0.004	0.002	0.002	0.002	0.003	0.003	0.003	0.003	0.003	0.005	0.000	0.000	
