# Supplementary material for: Novel attempt at discrimination of a bullet-shaped siphonophore (Family Diphyidae) using matrix-assisted laser desorption/ionization time of flight mass spectrometry (MALDI-ToF MS)
Source: Sci Rep. 2021 Sep 24;11:19077. doi: 10.1038/s41598-021-98724-z (PMC8463557; doi:10.1038/s41598-021-98724-z)
Supplement: Supplementary file 14 — Supplementary Information 14. [file 41598_2021_98724_MOESM14_ESM.pdf]

Table S5. K2P genetic distances of 25 ITS regions sequences between Diphyidae species in this study. Standard error estimates are shown above the diagonal in italics.

ITS regions	1	2	3	4	5	6	7	8	9	10	11	12	13	14	15	16	17	18	19	20	21	22	23	24	25
1. *Hippopodius_hippopus*_KE1910_Eddy3_HH1		0.022	0.021	0.021	0.021	0.018	0.022	0.023	0.022	0.022	0.022	0.022	0.022	0.023	0.023	0.023	0.021	0.021	0.021	0.022	0.022	0.021	0.024	0.024	0.024
2. *Chelophyes_appendiculata*_KE1910_Eddy3_CA1	0.167		0.007	0.007	0.007	0.021	0.014	0.014	0.014	0.014	0.014	0.014	0.014	0.014	0.014	0.014	0.011	0.011	0.011	0.012	0.012	0.016	0.024	0.024	0.024
3. *Chelophyes_contorta*_KC2005_S09_CC1	0.164	0.024		0.002	0.002	0.021	0.013	0.013	0.013	0.013	0.013	0.013	0.013	0.013	0.014	0.014	0.009	0.009	0.009	0.011	0.011	0.016	0.023	0.023	0.023
4. *Chelophyes_contorta*_KC2005_S09_CC2	0.161	0.021	0.002		0.000	0.021	0.013	0.013	0.013	0.013	0.013	0.013	0.013	0.013	0.013	0.013	0.009	0.009	0.009	0.011	0.011	0.016	0.024	0.024	0.024
5. *Chelophyes_contorta*_KC2005_S09_CC3	0.161	0.021	0.002	0.000		0.021	0.013	0.013	0.013	0.013	0.013	0.013	0.013	0.013	0.013	0.013	0.009	0.009	0.009	0.011	0.011	0.016	0.024	0.024	0.024
6. *Dimophyes_arctica*_KE1910_St0_DA1	0.124	0.160	0.160	0.157	0.157		0.020	0.020	0.020	0.020	0.020	0.020	0.020	0.020	0.021	0.021	0.020	0.020	0.020	0.022	0.022	0.019	0.023	0.022	0.022
7. *Diphyes_bojani*_KE1808_St9_DB1	0.170	0.089	0.076	0.073	0.073	0.149		0.002	0.008	0.008	0.008	0.008	0.008	0.007	0.008	0.008	0.012	0.012	0.012	0.014	0.014	0.018	0.023	0.022	0.022
8. *Diphyes_bojani*_KE1808_St9_DB2	0.173	0.092	0.078	0.076	0.076	0.149	0.002		0.008	0.008	0.008	0.008	0.008	0.007	0.007	0.007	0.013	0.013	0.013	0.014	0.014	0.018	0.023	0.023	0.023
9. *Diphyes_chamissonis*_DB1809_St10_DC1	0.176	0.084	0.071	0.068	0.068	0.152	0.028	0.026		0.000	0.000	0.000	0.000	0.004	0.005	0.005	0.013	0.013	0.013	0.014	0.014	0.018	0.024	0.023	0.023
10. *Diphyes_chamissonis*_DB1809_St11_DC2	0.176	0.084	0.071	0.068	0.068	0.152	0.028	0.026	0.000		0.000	0.000	0.000	0.004	0.005	0.005	0.013	0.013	0.013	0.014	0.014	0.018	0.024	0.023	0.023
11. *Diphyes_chamissonis*_DB1809_St11_DC3	0.176	0.084	0.071	0.068	0.068	0.152	0.028	0.026	0.000	0.000		0.000	0.000	0.004	0.005	0.005	0.013	0.013	0.013	0.014	0.014	0.018	0.024	0.023	0.023
12. *Diphyes_chamissonis*_DB1809_St11_DC4	0.176	0.084	0.071	0.068	0.068	0.152	0.028	0.026	0.000	0.000	0.000		0.000	0.004	0.005	0.005	0.013	0.013	0.013	0.014	0.014	0.018	0.024	0.023	0.023
13. *Diphyes_chamissonis*_DB1809_St11_DC5	0.176	0.084	0.071	0.068	0.068	0.152	0.028	0.026	0.000	0.000	0.000	0.000		0.004	0.005	0.005	0.013	0.013	0.013	0.014	0.014	0.018	0.024	0.023	0.023
14. *Diphyes_dispar*_KE1710_St11.5_DD1	0.176	0.086	0.076	0.073	0.073	0.152	0.024	0.021	0.007	0.007	0.007	0.007	0.007		0.002	0.002	0.013	0.013	0.013	0.015	0.015	0.018	0.023	0.023	0.023
15. *Diphyes_dispar*_KE1710_St11.5_DD2	0.179	0.089	0.079	0.076	0.076	0.155	0.026	0.024	0.009	0.009	0.009	0.009	0.009	0.002		0.000	0.013	0.013	0.013	0.015	0.015	0.018	0.024	0.024	0.024
16. *Diphyes_dispar*_KE1710_St11.5_DD3	0.179	0.089	0.079	0.076	0.076	0.155	0.026	0.024	0.009	0.009	0.009	0.009	0.009	0.002	0.000		0.013	0.013	0.013	0.015	0.015	0.018	0.024	0.024	0.024
17. *Eudoxoides_mitra*_KC2005_S12_EM1	0.156	0.048	0.038	0.036	0.036	0.149	0.071	0.073	0.071	0.071	0.071	0.071	0.071	0.074	0.076	0.076		0.000	0.000	0.012	0.012	0.017	0.026	0.026	0.026
18. *Eudoxoides_mitra*_KC2005_S12_EM2	0.156	0.048	0.038	0.036	0.036	0.149	0.071	0.073	0.071	0.071	0.071	0.071	0.071	0.074	0.076	0.076	0.000		0.000	0.012	0.012	0.017	0.026	0.026	0.026
19. *Eudoxoides_mitra*_KC2005_S12_EM3	0.156	0.048	0.038	0.036	0.036	0.149	0.071	0.073	0.071	0.071	0.071	0.071	0.071	0.074	0.076	0.076	0.000	0.000		0.012	0.012	0.017	0.026	0.026	0.026
20. *Eudoxoides_spiralis*_KE1808_St8_ES1	0.164	0.063	0.053	0.051	0.051	0.170	0.079	0.081	0.089	0.089	0.089	0.089	0.089	0.092	0.094	0.094	0.056	0.056	0.056		0.000	0.018	0.026	0.026	0.026
21. *Eudoxoides_spiralis*_KE1910_Eddy3_ES2	0.164	0.063	0.053	0.051	0.051	0.170	0.079	0.081	0.089	0.089	0.089	0.089	0.089	0.092	0.094	0.094	0.056	0.056	0.056	0.000		0.018	0.026	0.026	0.026
22. *Lensia_cossack*_KE1910_Eddy1_LC1	0.152	0.097	0.105	0.102	0.102	0.127	0.121	0.121	0.118	0.118	0.118	0.118	0.118	0.116	0.118	0.118	0.111	0.111	0.111	0.124	0.124		0.021	0.021	0.021
23. *Muggiaea_atlantica*_DB1804_St14_MA1	0.194	0.198	0.191	0.195	0.195	0.190	0.183	0.186	0.189	0.189	0.189	0.189	0.189	0.183	0.187	0.187	0.212	0.212	0.212	0.226	0.226	0.161		0.002	0.002
24. *Muggiaea_atlantica*_DB1804_St14_MA2	0.191	0.195	0.188	0.191	0.191	0.187	0.179	0.183	0.186	0.186	0.186	0.186	0.186	0.180	0.183	0.183	0.209	0.209	0.209	0.223	0.223	0.158	0.002		0.000
25. *Muggiaea_atlantica*_DB1804_St14_MA3	0.191	0.195	0.188	0.191	0.191	0.187	0.179	0.183	0.186	0.186	0.186	0.186	0.186	0.180	0.183	0.183	0.209	0.209	0.209	0.223	0.223	0.158	0.002	0.000	
